# A20 regulates lymphocyte adhesion in murine neuroinflammation by restricting endothelial ICOSL expression in the CNS

**DOI:** 10.1172/JCI168314

**Published:** 2023-12-15

**Authors:** Lisa Johann, Sasha Soldati, Kristin Müller, Josephine Lampe, Federico Marini, Matthias Klein, Eva Schramm, Nathalie Ries, Carsten Schelmbauer, Ilaria Palagi, Khalad Karram, Julian C. Assmann, Mahtab A. Khan, Jan Wenzel, Mirko H.H. Schmidt, Jakob Körbelin, Dirk Schlüter, Geert van Loo, Tobias Bopp, Britta Engelhardt, Markus Schwaninger, Ari Waisman

**Affiliations:** 1 Institute for Molecular Medicine, University Medical Center of the Johannes Gutenberg, University Mainz, Mainz, Germany.; 2 Theodor Kocher Institute, University of Bern, Bern, Switzerland.; 3 Institute for Experimental and Clinical Pharmacology and Toxicology, Center of Brain, Behavior and Metabolism (CBBM), University of Lübeck, Lübeck, Germany.; 4 DZHK (German Research Centre for Cardiovascular Research), Hamburg-Lübeck-Kiel, Germany.; 5 Institute of Medical Biostatistics, Epidemiology and Informatics (IMBEI),; 6 Research Center for Immunotherapy (FZI), and; 7 Institute for Immunology, University Medical Center of the Johannes Gutenberg, University Mainz, Mainz, Germany.; 8 Institute of Anatomy, Medical Faculty Carl Gustav Carus, Technische Universität Dresden School of Medicine, Dresden, Germany.; 9 University Medical Center Hamburg-Eppendorf, Department of Oncology, Hematology and Bone Marrow Transplantation, Hamburg, Germany.; 10 Hannover Medical School, Institute of Medical Microbiology and Hospital Epidemiology, Hannover, Germany.; 11 Department of Biomedical Molecular Biology, Ghent University, Ghent, Belgium.; 12 VIB-UGent Center for Inflammation Research, Ghent, Belgium.

**Keywords:** Autoimmunity, Neuroscience, Endothelial cells, Mouse models, Multiple sclerosis

## Abstract

A20 is a ubiquitin-modifying protein that negatively regulates NF-κB signaling. Mutations in A20/*TNFAIP3* are associated with a variety of autoimmune diseases, including multiple sclerosis (MS). We found that deletion of A20 in central nervous system (CNS) endothelial cells (ECs) enhances experimental autoimmune encephalomyelitis (EAE), a mouse model of MS. A20^ΔCNS-EC^ mice showed increased numbers of CNS-infiltrating immune cells during neuroinflammation and in the steady state. While the integrity of the blood-brain barrier (BBB) was not impaired, we observed a strong activation of CNS-ECs in these mice, with dramatically increased levels of the adhesion molecules ICAM-1 and VCAM-1. We discovered ICOSL to be expressed by A20-deficient CNS-ECs, which we found to function as adhesion molecules. Silencing of ICOSL in CNS microvascular ECs partly reversed the phenotype of A20^ΔCNS-EC^ mice without reaching statistical significance and delayed the onset of EAE symptoms in WT mice. In addition, blocking of ICOSL on primary mouse brain microvascular ECs impaired the adhesion of T cells in vitro. Taken together, we propose that CNS EC-ICOSL contributes to the firm adhesion of T cells to the BBB, promoting their entry into the CNS and eventually driving neuroinflammation.

## Graphical abstract

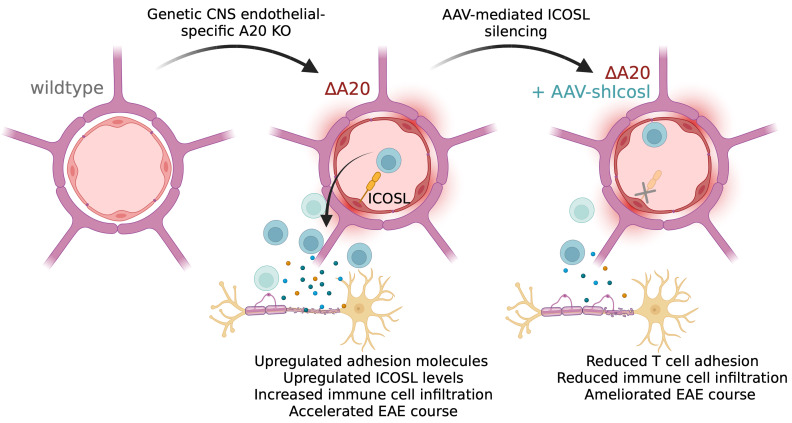

## Introduction

Multiple sclerosis (MS) is the most prevalent chronic inflammatory disease of the central nervous system (CNS) in young adults (1). To date, the causes of MS are not fully understood, yet it is presumed to be an autoimmune disease driven by autoreactive T cells targeting CNS antigens (2). Upon reactivation at the CNS borders, autoreactive immune cells infiltrate the CNS parenchyma and attack the myelin sheaths that surround neurons, eventually leading to axonal loss and causing neuroinflammation (3, 4).

Transmigration of T cells across the blood-brain barrier (BBB) represents a key step in the pathology of MS and its animal model, experimental autoimmune encephalomyelitis (EAE) (5), which follows a multistep process involving interaction of different adhesion molecules with integrins (6, 7). While expression of these molecules on the BBB is usually low, proinflammatory cytokine signaling during neuroinflammation induces dramatic brain microvascular endothelial cell (BMEC) activation (8–12). IL-1 signaling is an essential pathway driving the expression of adhesion molecules like intercellular adhesion molecule 1 (ICAM-1) and vascular cell adhesion molecule 1 (VCAM-1) on BMECs through nuclear translocation of nuclear factor κ-light-chain-enhancer of activated B cells (NF-κB), thus promoting the firm adhesion of leukocytes to the BBB (13–18).

A20 is an integral molecule for the negative regulation of the NF-κB signaling pathway. The transcription factor NF-κB is active in BMECs in a variety of conditions associated with vascular dysfunction and BBB impairment (19–21). Moreover, a protective role was ascribed to the NF-κB essential modulator (NEMO) and the upstream kinase TAK1 in BMECs, as their deletion causes BBB disruption and BMEC cell death partly mediated by TNF signaling (22). Importantly, A20 is well known to counteract the cytotoxic effects of TNF (23–25).

Genetic variations in the *TNFAIP3* gene, encoding for A20, have been linked to susceptibility to MS (26). Interestingly, mice deficient for A20 develop a spontaneous neuroinflammation with remarkable micro- and astrogliosis as well as BMEC activation (27). Deletion of A20 specifically in astrocytes or microglia further proved the protective function of A20 in the establishment of active EAE and spontaneous neuroinflammation (28–30). Besides glial cells, endothelial cells (ECs) also express high levels of A20 in the steady state condition (31), yet studies regarding the role of A20 in CNS-ECs are, so far, lacking.

We found that mice lacking A20 in CNS-ECs develop an exacerbated EAE disease upon adoptive transfer (AT) of encephalitogenic T cells. While we did not observe pronounced alterations in the structure of the microvasculature or BBB integrity, we found that loss of A20 in CNS-ECs dramatically upregulated ICAM-1, VCAM-1 and the inducible T cell costimulator ligand (ICOSL). Using a CNS-microvasculature endothelial cell–specific viral vector to silence ICOSL, we could partly rescue the more severe AT-EAE course in A20^ΔCNS-EC^ mice and delay the onset of an active EAE disease in WT mice. Mechanistically, we demonstrated that ICOSL drove the adhesion of Th1 and Th17 cells to an in vitro BBB model. Taken together, our work reveals A20-regulated ICOSL as an adhesion molecule involved in the multi-step transmigration process of T cells across the BBB under inflammatory conditions.

## Results

### Mice with A20-deficient CNS-ECs are hypersensitive to AT EAE.

**Figure 1:**
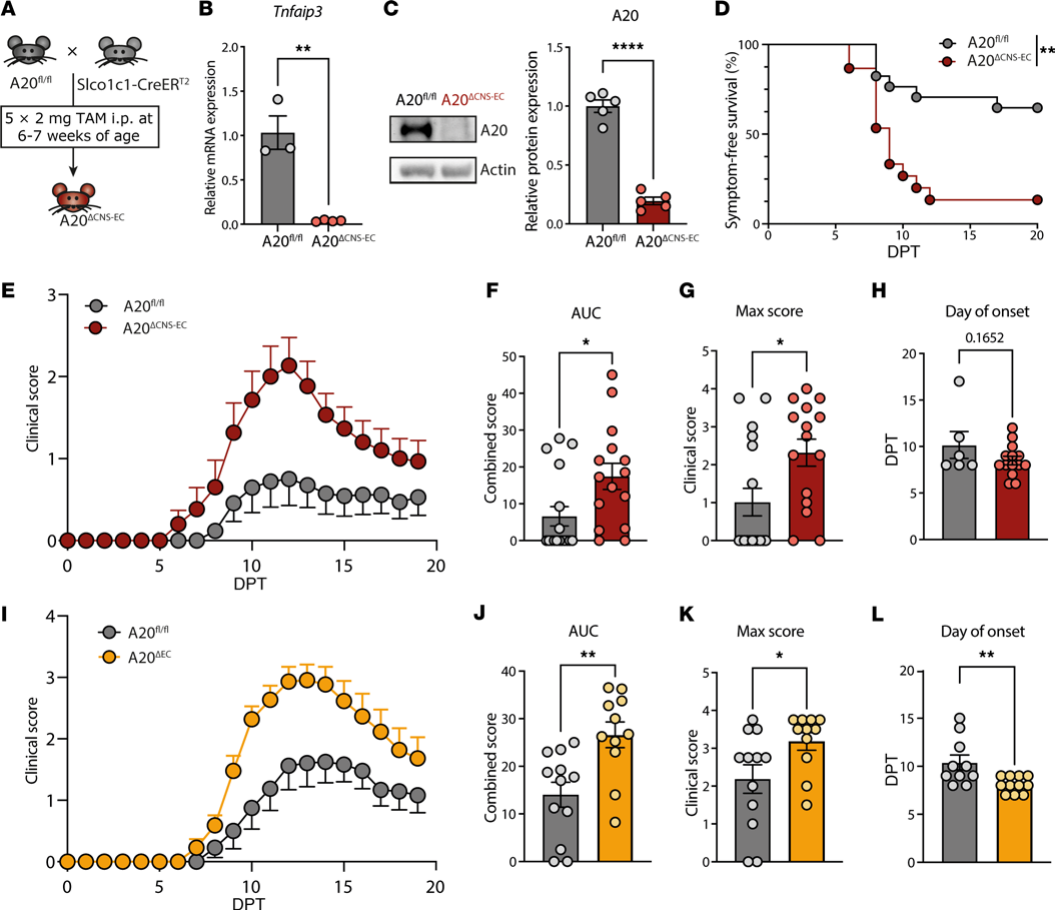
CNS EC-A20 plays a protective role in CNS autoimmunity. (**A**) Breeding strategy for generation of A20^ΔCNS-EC^ mice. Conditional deletion of *Tnfaip3* in A20^ΔCNS-EC^ mice is achieved through TAM injections. (**B**) Validation of *Tnfaip3* deletion in pMBMECs from A20^ΔCNS-EC^ mice by RT-PCR. *Tnfaip3* mRNA levels are presented relative to control (*n* = 3–4 mice per group). (**C**) Western blot analysis of A20 protein in cultured primary brain endothelial cells from A20^ΔCNS-EC^ and littermate control mice. Protein expression of A20 is normalized to actin levels and presented relative to the expression in controls (*n* = 5 mice per group). (**D**–**H**) Adoptive transfer-EAE (AT-EAE) disease in A20^ΔCNS-EC^ mice and A20^fl/fl^ littermates. Clinical signs of EAE were monitored daily. Data is pooled from 2 independent experiments with *n* = 15–17 mice per group. (**D**) Probability of symptom-free survival is shown as Kaplan Meier curve. (**E**) Clinical signs of EAE are shown as mean clinical disease scores ± SEM. (**F**) AUC, (**G**) maximum clinical scores, and (**H**) day of onset analyses of clinical course shown in (**E**). Every circle represents a single mouse. (**I**–**L**) AT-EAE disease was induced in A20^ΔEC^ lacking A20 in all endothelial cells driven by the Cdh5-CreER^T2^ and A20^fl/fl^ littermate controls as described before. Clinical signs of EAE were monitored daily. Data is pooled from 2 independent experiments with *n* = 11–12 mice per group. (**I**) Clinical signs of EAE are shown as mean clinical disease scores ± SEM. (**J**) AUC, (**K**) maximum clinical scores, and (**L**) day of onset analyses of clinical course shown in (**I**). Every circle represents a single mouse. Statistical significance was determined by 2-tailed unpaired Student’s *t* test (**B**, **C**, **F**–**H**, and **J**–**L**) or Log-rank (Mantel-Cox) test (**D**). *P<0.05, **P<0.01, ****P<0.0001.

To investigate the involvement of CNS-endothelial A20 in autoimmune neuroinflammation, we generated A20^ΔCNS-EC^ mice that lack A20 in CNS-ECs upon tamoxifen-inducible (TAM-inducible) Cre recombination (Figure 1A). RT-PCR and Western blot analyses of primary mouse brain microvascular endothelial cells (pMBMECs) confirmed the deletion of *Tnfaip3*/A20 (Figure 1, B and C). To our surprise, we did not observe a difference in disease severity between A20^ΔCNS-EC^ mice and littermate controls in 3 different active EAE models where mice were immunized with MOG_35–55_ in complete Freund’s adjuvant (CFA) (Supplemental Figure 1, A–L; supplemental material available online with this article; https://doi.org/10.1172/JCI168314DS1). As CFA is known to activate CNS-ECs (13), we next studied the phenotype of A20^ΔCNS-EC^ mice in an AT-EAE model that circumvents CFA-driven effects.

Interestingly, we found that, compared to littermate controls, the majority of A20^ΔCNS-EC^ mice developed EAE symptoms (Figure 1D). This resulted in an increased average disease severity, with increased AUC values and maximum scores (Figure 1, E–G). A20^ΔCNS-EC^ mice, further, showed a tendency toward earlier disease onset (Figure 1H). To exclude astrocytic contamination, which was previously reported for the Slco1c1-CreER^T2^ mouse line (32) we generated mice lacking A20 in all endothelial cells (A20^ΔEC^) by crossing A20^fl/fl^ mice to Cdh5(PAC)-CreER^T2^ mice (33) to validate our data. Indeed, we observed a very similar phenotype with an overall increased disease severity (Figure 1, I–K). A20^ΔEC^ mice, further, showed a significantly accelerated disease onset, with mice developing symptoms, on average, 2 days earlier than controls (Figure 1L).

We next focused on a different model involving CNS inflammation and used an occlusion of the middle cerebral artery *(*MCAO) model to induce stroke in A20^ΔCNS-EC^ mice. We observed significantly increased infarct and edema volumes, overall leading to a mildly increased edema-corrected infarct volume in A20^ΔCNS-EC^ mice (Supplemental Figure 2). Together, these data strongly suggest a protective role for CNS EC-A20 in CNS autoimmunity and sterile inflammation.

### Loss of A20 in CNS-ECs drives immune cell infiltration into the CNS.

**Figure 2:**
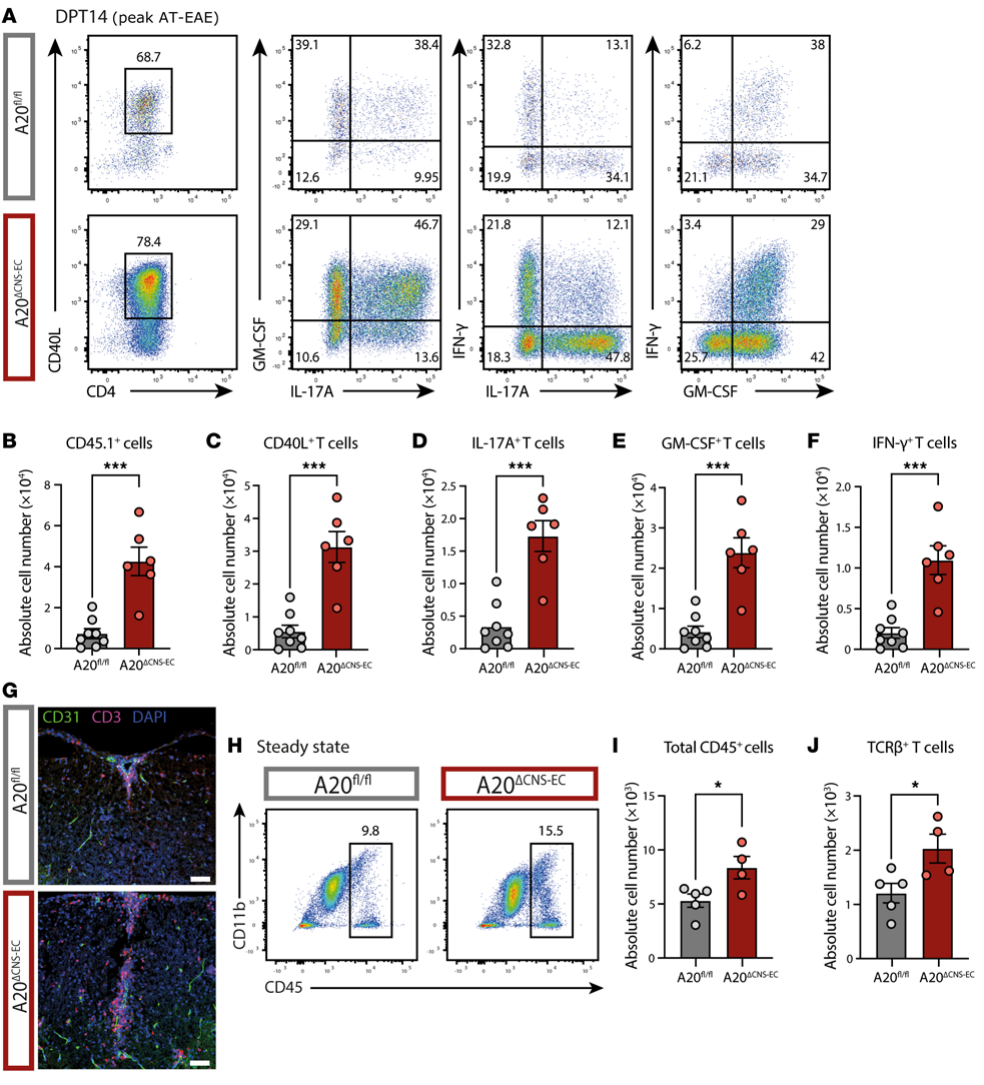
Loss of CNS EC-A20 drives T cell infiltration during EAE and in the steady state. (**A**–**E**) AT-EAE disease was induced in A20^ΔCNS-EC^ mice and A20^fl/fl^ littermate controls. At the peak of the disease (DPT 14) SC-infiltrating CD45.1^+^ transferred T cells were isolated and analyzed by flow cytometry after a MOG recall assay ex vivo. (**A**) Representative gating strategy for CD40L^+^ CD4^+^ T cells and within this population, frequencies of GM-CSF, IL-17A, IFN-γ and double-producing cells; pregated as single, live, CD45.1^+^ transferred T cells. (**B**–**F**) Quantification of immune cell populations. Data is representative for 2 independent experiments with *n* = 6–8 mice per group. (**B**) Absolute cell numbers of CD45.1^+^ transferred T cells, (**C**) CD40L^+^ among transferred T cells, (**D**) IL-17A^+^, (**E**) GM-CSF^+^ and (**F**) IFN-γ^+^ cells among CD40L^+^ cells. (**G**) Representative immunostainings of CD3^+^ T cells (magenta), CD31^+^ endothelial cells (green) and nuclear staining (DAPI, blue) in the SC at the peak of AT-EAE (DPT14) of A20^ΔCNS-EC^ and A20^fl/fl^ mice (scale bar: 50 μm). (**H**–**J**) Flow cytometric analysis of CNS-infiltrating cells in the steady state of A20^ΔCNS-EC^ and A20^fl/fl^ mice 1 week after TAM treatment. Cells were isolated from pooled SC and brain tissue. (**H**) Representative gating strategy for CD45^+^ total immune cells, pregated as single, live cells. (**I** and **J**) Quantification of immune cell populations. Data is representative for 3 independent experiments with *n* = 4–5 mice per group. (**I**) Absolute cell numbers of CD45^+^ total infiltrates and, among them, (**J**) number of TCRβ^+^ T cells. Statistical significance was determined by 2-tailed unpaired Student’s *t* test. * p<0.05, *** p<0.001.

To investigate the extent of leukocyte infiltration, we isolated immune cells from the SC at the peak of AT-EAE disease and analyzed cell subsets and cytokine production by flow cytometry after antigen recall (Figure 2A). We found dramatically increased numbers of CD45.1^+^ transferred, and, within the transferred cells, also increased numbers of CD40L^+^ MOG_35–55_ -specific T cells in A20^ΔCNS-EC^ mice (Figure 2, B and C). Also, the number of cytokine-producing cells was significantly increased (Figure 2, D–F). Furthermore, absolute cell counts of dendritic cells, neutrophils, and monocytes were elevated in A20^ΔCNS-EC^ mice (Supplemental Figure 3, A–E). We next analyzed the location of CD3^+^ T cells in the SC by immunostaining. Compared with controls, we found high numbers of T cells in the SC of A20^ΔCNS-EC^ mice, with cells forming perivascular cuffs but also penetrating deep into the parenchyma (Figure 2G). As EAE disease severity is strongly dependent on the ratio of effector to regulatory T cells (Treg cells) (34), we next assessed if loss of A20 in CNS-ECs influenced Treg cell frequencies. We did not observe changes in Treg cell frequencies or effector T cell to Treg cell ratio at day posttransfer (DPT) 18 (Supplemental Figure 4, A–D). Based on these observations, we conclude that loss of A20 in CNS-ECs promoted immune cell infiltration across the BBB under inflammatory conditions without affecting the proportion of effector T cells to Treg cells.

We next addressed the question of whether loss of A20 in CNS-ECs caused infiltration of immune cells even in the absence of exogenously induced inflammation. We assessed the numbers of CD45^+^ total infiltrates, TCRβ^+^ T cells and CD11b^+^ myeloid cells in the steady state CNS a week after TAM injection. Indeed, we found increased numbers of immune cells infiltrating the CNS in A20^ΔCNS-EC^ mice in the steady state compared with littermate controls (Figure 2, H–J, and Supplemental Figure 3, F and G). Interestingly, the frequencies of TCRβ^+^ T cells and CD11b^+^ myeloid cells among all infiltrating cells were not altered (Supplemental Figure 3H). Taken together, these data suggest that the loss of A20 in CNS-ECs provokes the infiltration of immune cells across the BBB even in the absence of an inflammatory stimulus.

### A20 protects from excessive adhesion molecule expression in steady state CNS-ECs.

**Figure 3:**
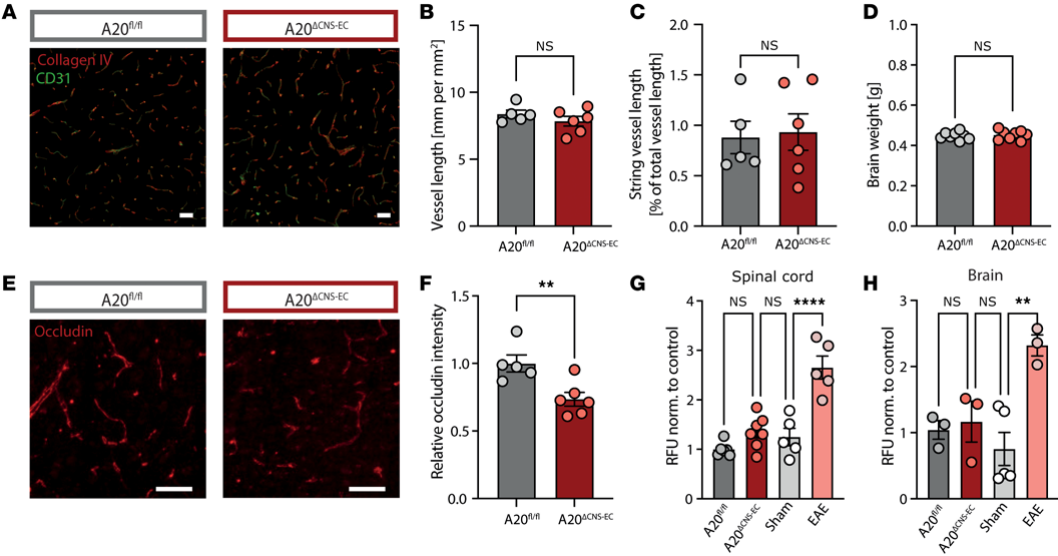
A20-deficiency in CNS-ECs does not impair BBB integrity. (**A**–**C**) Brain sections from A20^ΔCNS-EC^ and A20^fl/fl^ mice were stained for collagen IV (red) and CD31 (green) to determine vessel length and string vessel length. String vessels were identified as capillaries that have lost CD31-positive endothelial cells and only consist of the basement membrane protein collagen IV. Representative microscopic images are shown in (**A**). Scale bar: 50 μm. (**B**) Vessel length (in mm per mm^2^) and (**C**) string vessel length (%) normalized to A20^fl/fl^ littermate controls were quantified (*n* = 5–6 mice per group). (**D**) Brain weight of A20^ΔCNS-EC^ and A20^fl/fl^ mice (*n* = 8 mice per group) shown as representative of 2 individual cohorts. (**E** and **F**) Brain sections from A20^ΔCNS-EC^ and A20^fl/fl^ mice were stained for occludin (red). Representative microscopic images are shown in (**E**). Scale bar: 50 μm. (**F**) Occludin intensity was quantified and is presented relative to A20^fl/fl^ littermate control mice (*n* = 5–6 mice per group) as representative of 2 individual cohorts. (**G** and **H**) A20^ΔCNS-EC^ and A20^fl/fl^ mice were injected with 2 mM 3-5 kDa FITC-dextran in PBS i.p. After 15 minutes, SC and brain were isolated and homogenized in PBS. Fluorescence was measured in the supernatant and raw fluorescence units (RFU) were normalized to tissue weight. PBS-injected mice were used as sham controls; mice at the peak of an active EAE (scores between 1.5 and 3.5) were used as positive controls. Normalized RFUs are shown for SC (**G**) and brain (**H**) relative to A20^fl/fl^ controls (SC: *n* = 5–7; brain: *n* = 3–5 mice per group). Statistical significance was determined by 2-tailed unpaired Student’s *t* test (**B**–**D** and **F**) or ordinary 1-way ANOVA with Tukey’s multiple comparisons test (**G** and **H**). **P<0.01, ****P<0.0001.

To test whether deficiency for A20 in BMECs results in CNS blood vessel abnormalities, we performed immunostainings of the endothelial cell marker CD31 and of collagen IV as an integral basement membrane component. We observed neither alterations in vessel length nor increased numbers of empty basement membrane strands, also known as string vessels (35), in the brains of A20^ΔCNS-EC^ mice (Figure 3, A–C). We next focused on BBB integrity in A20^ΔCNS-EC^ mice. By performing immunostaining for the tight junction molecule occludin, we observed reduced fluorescence intensity in BMECs of A20^ΔCNS-EC^ mice (Figure 3, E and F). However, brain weight, as an indicator of brain edema, was not increased compared with controls (Figure 3D). To exclude a disruption of the BBB and the blood-spinal cord barrier (BSCB), we assessed integrity by injecting a small 3–5 kDa dextran tracer and measuring fluorescence in brain or SC. Compared with mice at the peak of an active EAE disease, in which BBB and BSCB integrity are compromised, A20^ΔCNS-EC^ mice did not show an increased permeability for FITC-dextran, confirming normal BBB and BSCB integrities (Figure 3, G and H).

**Figure 4:**
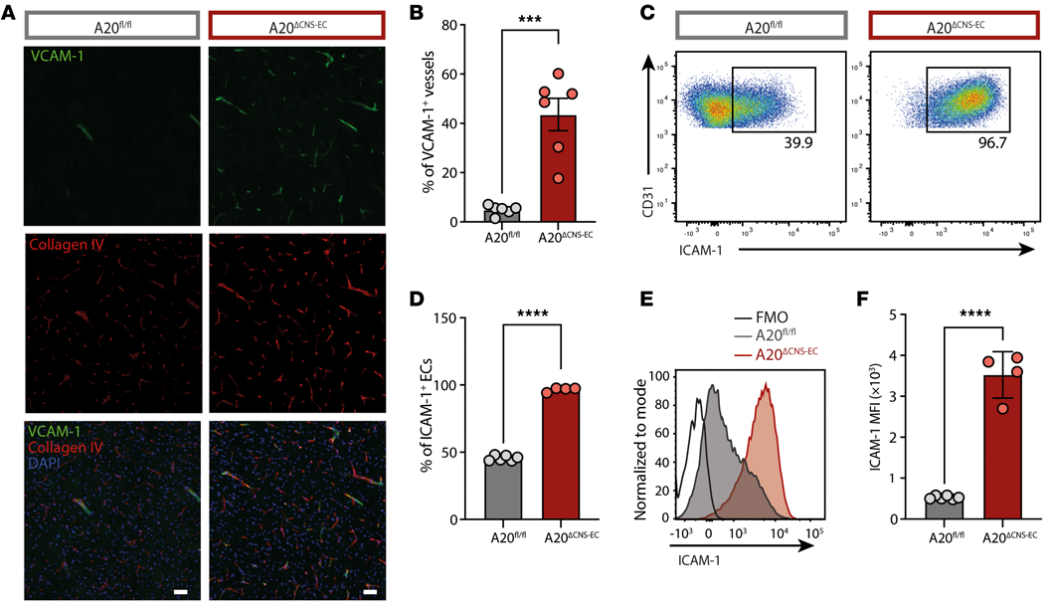
A20-deficiency in CNS-ECs drives adhesion molecule expression. (**A** and **B**) Brain sections were stained for VCAM-1 (green) and collagen IV (red) together with a nuclear staining (DAPI, blue). Representative microscopic images are shown in **A**. Scale bar: 50 μm. (**B**) Frequency of VCAM-1^+^ vessels quantified as percentage of all collagen IV positive vessels (*n* = 6 mice per group). (**C**–**F**) Flow cytometric analysis of ICAM-1 on CNS-ECs isolated from pooled brain and spinal cord tissue from A20^ΔCNS-EC^ and A20^fl/fl^ littermate control mice. Data is representative from 3 individual experiments with *n* = 4–6 mice per group. (**C**) Representative gating strategy for ICAM-1^+^ CNS-ECs; gate was set based on fluorescence minus 1 (FMO) control. CNS-ECs were pregated as single, live, CD45^–^ CD11b^–^ Ly6C^+^ CD31^+^ cells. (**D**) Frequency of ICAM-1^+^ ECs quantified as percentage of all CNS-ECs. (**E**) Histogram of ICAM-1 fluorescence on ECs in A20^ΔCNS-EC^, A20^fl/fl^ mice and FMO control. (**F**) Mean fluorescence intensity (MFI) presented as geometric mean of ICAM-1 on CNS-ECs. Statistical significance was determined by 2-tailed unpaired Student’s *t* test (**B**, **D**, and **F**). ***P<0.001, ****P<0.0001.

As T cell transmigration across the BBB is mainly dependent on the surface expression levels of adhesion molecules (36), we next investigated whether the increased numbers of immune cells infiltrating the CNS in A20^ΔCNS-EC^ mice were due to alterations in adhesion molecule expression. By performing immunostaining of VCAM-1 together with collagen IV, we detected dramatically increased numbers of VCAM-1^+^ microvessels in the brain cortex of A20^ΔCNS-EC^ mice (Figure 4, A and B). Also, ICAM-1 levels were dramatically increased on CNS microvessels in A20^ΔCNS-EC^ mice, with almost 100% of CNS-ECs expressing ICAM-1 (Figure 4, C–F). Both molecules are well known to mediate the multistep process of immune cell transmigration across the BBB (6). Our data suggest that loss of A20 in CNS-ECs does not influence BBB integrity but causes a dramatic upregulation of cell adhesion molecules, which at least partially drives leukocyte infiltration and AT-EAE severity.

### RNA-Seq reveals strong activation of CNS-ECs in A20^ΔCNS-EC^ mice.

**Figure 5:**
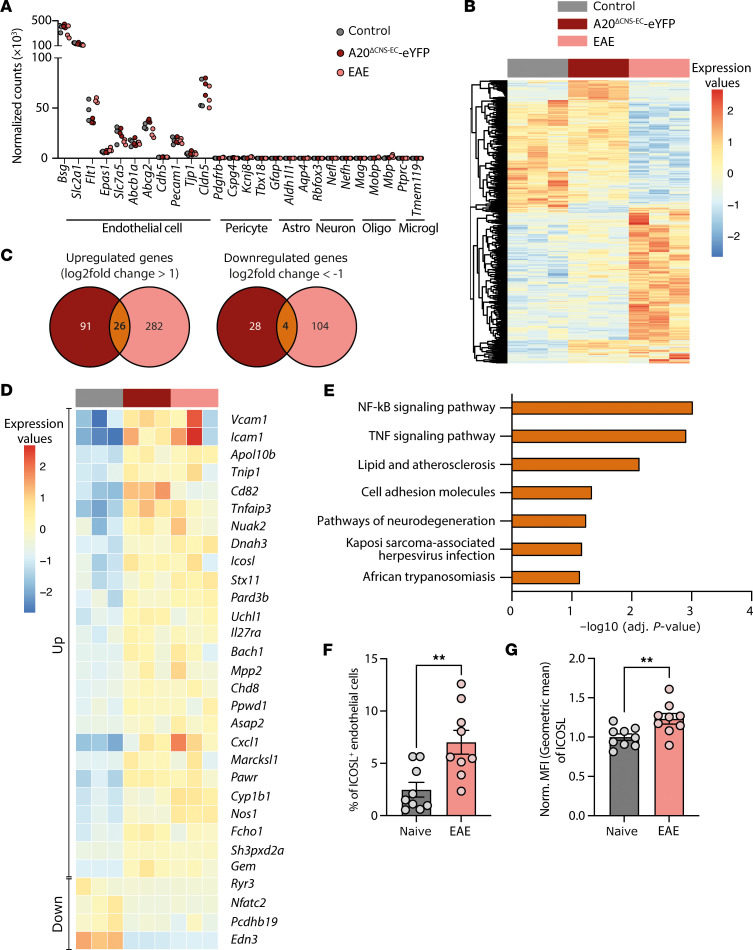
RNA-Seq identifies A20-regulated ICOSL as potential adhesion molecule. (**A**–**E**) RNA-Seq of sorted CNS-ECs from SC of naive A20^ΔCNS-EC^-eYFP (red), A20^fl/fl^ littermate control mice (referred to as control, grey), and A20^fl/fl^ mice at day 10 after EAE immunization with MOG_35–55_/CFA and PTx (referred to as EAE, pink) (*n* = 3 mice per group). (**A**) Purity of EC sorting was assessed by plotting normalized counts for marker genes of ECs, pericytes, astrocytes (astro), neurons, oligodendrocytes (oligo), and microglia (microgl). (**B**) Heatmap showing color-coded standardized z-scores for the expression values of genes differentially regulated in A20^ΔCNS-EC^ or EAE CNS-ECs compared with control. Each column represents an individual mouse. (**C**) Venn diagrams showing the number of commonly upregulated (log_2_ fold change greater than 1) and commonly downregulated (log_2_ fold change less than –1) genes in A20^ΔCNS-EC^-eYFP and EAE mice compared with control. (**D**) Heatmap showing color-coded standardized z-scores for the expression values of the 30 commonly DE genes. Each column represents an individual mouse. (**E**) KEGG pathway analysis of the 30 commonly DE genes shown in (**D**). (**F** and **G**) ECs were isolated from the CNS of WT mice at day 10 postimmunization with MOG_35–55_/CFA and PTx or from naive mice and stained for flow cytometric analysis of ICOSL. Data is pooled from 3 individual experiments with *n* = 9 mice per group. (**F**) Percentage and (**G**) normalized geometric MFI of ICOSL among all ECs were quantified. Statistical significance was determined by 2-tailed unpaired Student’s *t* test. **P<0.01.

Blocking immune cell infiltration by targeting α4-integrin, a binding partner of VCAM-1, with natalizumab is an effective treatment option for patients with MS presenting with a relapsing-remitting disease course (37–39). Yet, this therapeutic strategy comes with a risk of developing severe CNS infections (40). Identification of novel adhesion molecules facilitating the extravasation of specific cell subsets is thus essential. We therefore made use of the A20^ΔCNS-EC^ mice to characterize their CNS-EC adhesion molecule profile in depth by RNA-Seq. Crossing these mice with R26R-EYFP reporter mice allowed us to sort for CNS-ECs from the SC with Cre-mediated recombination, which could be observed in approximately 90% of all ECs (Supplemental Figure 5, A and B). To focus on molecules involved in immune cell transmigration, we included a control group of WT-like A20^fl/fl^ mice at DPI 10 of an active EAE disease, a time point where immune cell extravasation across the BBB is known to happen (41). We first confirmed the purity of the sorted SC-ECs by plotting endothelial marker genes as well as genes specific for other CNS-resident cells (Figure 5A). We next focused on genes that were differentially expressed (DE) in either the A20^ΔCNS-EC^-eYFP or the EAE group compared with the control condition, respectively. While most of the DE genes were found in the EAE situation, a substantial amount of DE genes could also be identified in A20^ΔCNS-EC^-eYFP mice (Figure 5B). The overlap of DE genes in the A20^ΔCNS-EC^-eYFP and the EAE condition resulted in 26 genes that were commonly upregulated and 4 genes that were commonly downregulated (Figure 5, C and D). Among these genes, *Icam1* and *Vcam1* represented the top upregulated genes, confirming our previous findings. Also, *Tnfaip3* itself was among the upregulated genes, yet mapping the reads of *Tnfaip3* to the genetic locus confirmed the excision of exon 3 in A20^ΔCNS-EC^ mice, rendering the transcript nontranslatable (Supplemental Figure 5C) (42). To investigate if other genes related to immune cell transmigration across the BBB were amongst the commonly DE genes, we performed a KEGG pathway analysis. The most prominent pathways were the inflammatory *NF-*κ*B* and *TNF* signaling pathways, pointing out the essential function of A20 as negative regulator of these signaling cascades. Interestingly, and as hypothesized, also the cell adhesion molecules pathway was amongst the top KEGG terms (Figure 5E). This was further underscored by a Gene Ontology (GO) term analysis, in which leukocyte cell-cell adhesion, membrane to membrane docking, and cell adhesion represented the top affected biological processes (Supplemental Figure 5D).

### ICOSL is upregulated on CNS-ECs under inflammatory conditions.

Interestingly, besides *Icam1* and *Vcam1,* *Icosl* was also identified as cell adhesion molecule in the KEGG pathway analysis, and, moreover, *Icosl* expression levels in A20^ΔCNS-ECs^ and DPI10 ECs were very similar to those of *Icam1* and *Vcam1* (Supplemental Figure 5E). RT-PCR validation of *Icosl* expression indeed confirmed its upregulation in sorted CNS-ECs from naive A20^ΔCNS-EC^ mice compared with littermate controls (Supplemental Figure 5F). In addition, we could validate the upregulation of ICOSL protein on CNS-ECs in vivo during EAE (Figure 5, F and G).

Expression of ICOSL on peripheral ECs as well as on the human BMEC-derived cell line hCMEC/D3 is strongly upregulated by various inflammatory stimuli in vitro (43, 44). We thus investigated *Icosl* mRNA levels in pMBMECs isolated from A20^ΔCNS-EC^ mice and found increased levels in response to TNF stimulation compared with unstimulated or TNF-stimulated A20^fl/fl^ control pMBMECs (Supplemental Figure 5G). Furthermore, while TNF stimulation of WT pMBMECs significantly increases *Icosl* expression, IL-1β stimulation led to an even higher upregulation of *Icosl* mRNA levels (Supplemental Figure 5H). Moreover, ICOSL upregulation in response to IL-1β stimulation followed a very similar pattern as ICAM-1 and VCAM-1 (Supplemental Figure 5I). These data indicate that, in addition to ICAM-1 and VCAM-1, ICOSL is also upregulated on CNS-ECs under inflammatory conditions and may therefore contribute to the onset of neuroinflammation.

### Silencing of CNS microvascular EC-ICOSL ameliorates AT-EAE in A20^ΔCNS-EC^ mice.

**Figure 6:**
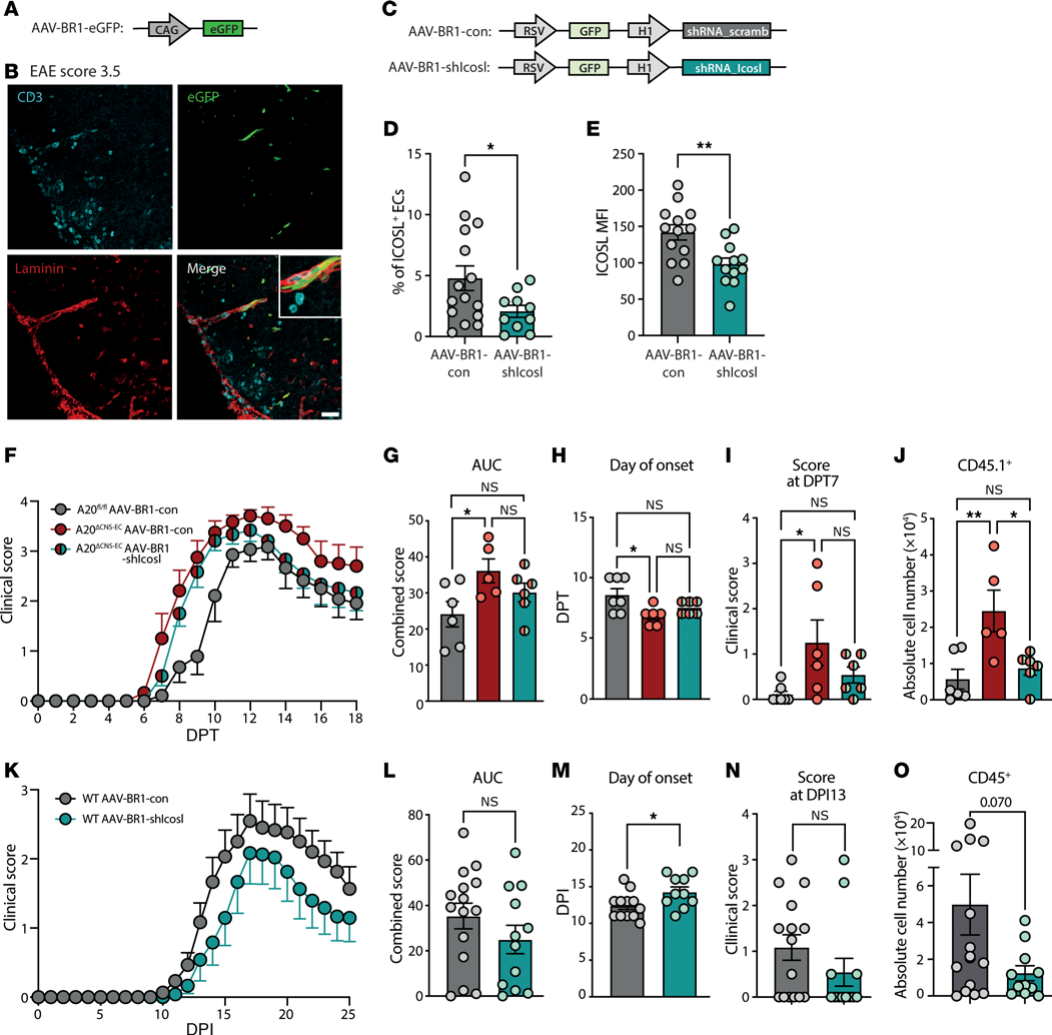
Knockdown of ICOSL on CNS microvascular ECs mildly ameliorates EAE severity. (**A**) Schematic representation of AAV-BR1-eGFP construct. (**B**) Representative immunofluorescence of SC tissue from mice treated with AAV-BR1-eGFP. Tissue was harvested at peak of EAE disease and stained for CD3 (cyan) and laminin (red). Endogenous eGFP is shown in green. Scale bar: 40 μm. (**C**) Schematic representation of AAV-BR1 constructs. (**D**) Percentage of ICOSL^+^ ECs and (**E**) MFI (geometric mean) of ICOSL on brain ECs in WT mice treated with AAV-BR1 constructs and 2 weeks later immunized with MOG/CFA. Analysis was performed at DPI10. Data is pooled from 2 individual experiments with *n* = 10–15 mice per group. (**F**–**J**) A20^ΔCNS-EC^ or A20^fl/fl^ controls were treated with AAV-BR1-shIcosl or AAV-BR1-con as indicated. After a week, mice were treated with TAM and AT-EAE was induced 4 weeks later (*n* = 5–7 mice per group). (**F**) Clinical signs were monitored daily and are shown as mean clinical scores ± SEM. (**G**) AUC, (**H**) day of onset, and (**I**) score at DPT7 analyses from data shown in **F**. (**J**) Flow cytometric analysis of CD4^+^ CD45.1^+^ transferred T cells in the SC at DPT18. (**K**–**O**) WT mice were injected with AAV-BR1 constructs. After 2 weeks, mice were immunized with MOG/CFA. (**K**) Clinical signs were monitored daily and are shown as mean clinical scores ± SEM. Data is pooled from 2 independent experiments with *n* = 12–15 mice per group. (**L**) AUC, (**M**) day of onset, and (**N**) score at day post immunization (DPI) 13 analyses of clinical course shown in **K**. (**O**) Flow cytometric analysis of CD45^+^ in the SC at DPI30. Statistical significance was determined by 2-tailed unpaired Student’s *t* test (**L**–**O**) or ordinary 1-way ANOVA with Tukey’s multiple comparisons test (**G**–**J**). *P<0.05, **P<0.01.

To evaluate the function of CNS EC-ICOSL in autoimmune neuroinflammation, we knocked down ICOSL by delivering shRNA to CNS microvascular ECs using the adeno-associated virus –BR1 (AAV-BR1). Because not 100% of CNS microvascular ECs are transduced upon AAV-BR1 application (45), we first confirmed that the location of the transduced ECs was in close proximity to the infiltrating immune cells during EAE. Indeed, we found multiple GFP^+^ vessels in close proximity to CD3^+^ T cells in the perivascular space, but also in close proximity to T cells infiltrating into the SC parenchyma in EAE mice treated with AAV-BR1-eGFP (Figure 6, A and B). Immunofluorescence further proved specificity of the AAV-BR1-eGFP in targeting vessels, as eGFP only colocalized with laminin but not cortical neuronal NeuN (Supplemental Figure 6A). Next, we designed a CNS microvascular EC-specific AAV carrying either an shRNA against *Icosl* (AAV-BR1-shIcosl) or a scrambled shRNA (AAV-BR1-con) (Figure 6C). Both constructs express a GFP reporter under the relatively weak RSV promoter, which was chosen to exclude potential GFP-induced cytotoxicity (46). Yet, GFP intensity was still strong enough to validate the target specificity. Upon injection of the AAV-BR1-shIcosl construct, GFP^+^ cells could only be found among the CD31^+^ Ly6C^+^ EC population, but not in other CNS-resident cells (Supplemental Figure 6, B–F). Transduction of pMBMECs with AAV-BR1-shIcosl further proved the efficiency of this virus to knockdown *Icosl,* as expression levels were reduced by approximately 70% in IL-1β stimulated pMBMECs (Supplemental Figure 6G). Flow cytometry of CNS-ECs upon in vivo administration of AAV-BR1-shIcosl further demonstrated a significant reduction in the mean fluorescence intensity of ICOSL as well as in the percentage of ICOSL^+^ ECs in mice after immunization with MOG_35–55_/CFA (Figure 6, D and E). Of note, treatment with AAV-BR1-shIcosl did not influence protein levels of ICAM-1 or VCAM-1 in CNS-ECs (Supplemental Figure 6, H and I).

We next used this system to knockdown ICOSL in CNS microvascular ECs in A20^ΔCNS-EC^ mice to evaluate the contribution of ICOSL to their increased EAE phenotype. Administration of AAV-BR1-shIcosl, but not AAV-BR1-con, mildly ameliorated the AT-EAE disease course including the AUC, the day of onset, and the score at DPT7, a time point shortly after disease onset (Figure 6, F–I). Furthermore, AAV-BR1-shIcosl administration significantly reduced the number of transferred CD45.1^+^ T cells infiltrating into the SC (Figure 6J). Thus, ICOSL participates in driving autoimmune neuroinflammation in A20^ΔCNS-EC^ mice and its knockdown reduces the infiltration of encephalitogenic cells into the CNS.

### Endothelial ICOSL influences day of active EAE onset in WT mice.

Next, we wanted to recapitulate our findings in WT mice in an active EAE model. For this, AAV-BR1-shIcosl was injected i.v. and 2 weeks later mice were immunized with MOG_35–55_ in CFA. Confirming our previous findings, we also detected a mildly ameliorated disease and a tendency toward reduced numbers of infiltrating immune cells in mice treated with AAV-BR1-shIcosl (Figure 6, K–O). More importantly, disease onset could be delayed by an average of 2 days in the AAV-BR1-shIcosl–treated group (Figure 6M). These findings confirm a functional role for CNS microvascular EC-ICOSL in driving autoimmune neuroinflammation, possibly by promoting the infiltration of pathogenic T cells.

### ICOSL promotes T cell adhesion to the BBB in vitro.

**Figure 7:**
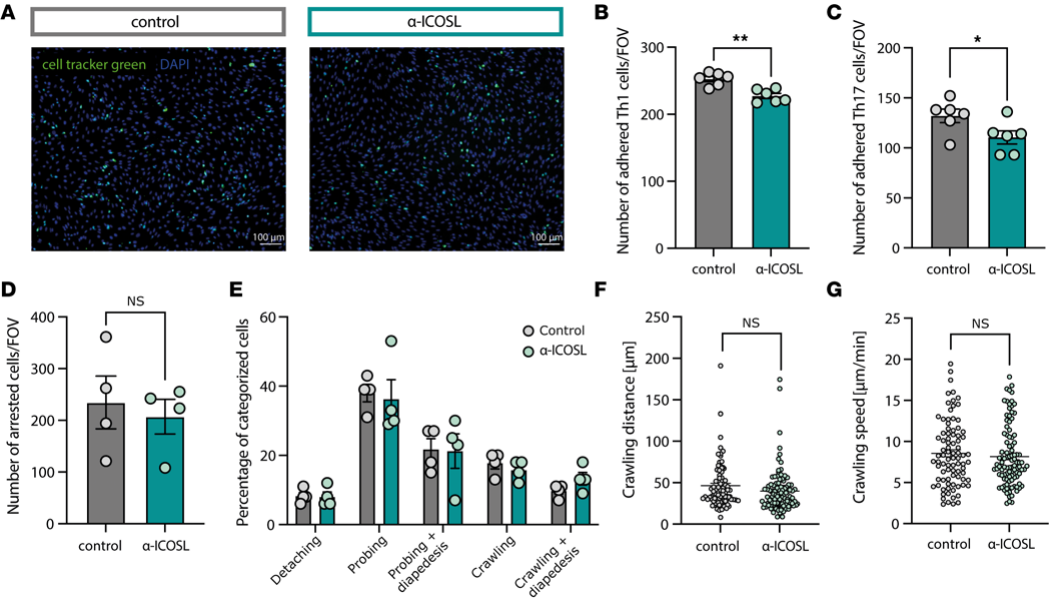
ICOSL regulates T cell adhesion to pMBMECs. (**A**–**C**) Static adhesion assay of T cells on pMBMECs. pMBMECs were stimulated with IL-1β for 72 hours and incubated for 30 minutes with α-ICOSL or control antibody. T cells were labelled with CellTracker Green CMFDA dye and added for 30 minutes to the pMBMECs on a shaking platform. After washing, images were taken and adhering cells were counted. Data is representative of 2 individual experiments with *n* = 6 per group. (**A**) Representative microscopic images of 2D2 Th1 cells (green) adhering to the pMBMEC monolayer (nuclear stain blue, DAPI). (**B**) Quantification of number of adhered 2D2 Th1 cells per field of view (FOV). (**C**) Quantification of number of adhered 2D2 Th17 cells per FOV. (**D**–**G**) Analysis of T cell postarrest behavior on pMBMECs. Data is pooled from 4 individual experiments. pMBMECs were stimulated with IL-1β for 72 hours and incubated for 30 minutes with α-ICOSL or control antibody. 2D2 Th1 T cell postarrest behavior was assessed by live cell imaging under physiological flow. (**D**) Number of arrested Th1 cells per FOV. (**E**) Quantification of T cell postarrest behavior. Each category is shown as fraction of the sum of the categorized cells. (**F**) Crawling distance of T cells that successfully completed diapedesis on IL-1β-stimulated isotype or α-ICOSL treated pMBMECs under flow conditions during 20 minutes observation time shown in μm. (**G**) Crawling speed of T cells that successfully completed diapedesis on IL-1β-stimulated isotype or α-ICOSL treated pMBMECs under flow conditions shown in μm/min. Data in **F** and **G** is pooled from 4 individual experiments with *n* = 86–102 tracks analyzed. Data was analyzed using 2-tailed unpaired Student’s *t* test (**B**–**D**, **F**, and **G**) or 2-way ANOVA with Šidák’s multiple comparison test (**E**). *P<0.05, **P<0.01.

ICOSL is an immunoglobulin superfamily member, expressing immunoglobulin-like domains that are known to mediate cell-cell adhesion through binding in a homophilic or heterophilic manner (47). ICOSL thus shares structural similarities with the known adhesion molecules ICAM-1, VCAM-1, and the more recently characterized molecules, activated leukocyte adhesion molecule (ALCAM-1), melanoma cell adhesion molecule (MCAM-1) and dual immunoglobulin-domain containing cell adhesion molecule (DICAM-1) (48–52). Recently, it was shown that ICOSL can bind to α_V_β_3_, an integrin known to be important for EAE development by mediating the recruitment of encephalitogenic T cells to the CNS (53, 54). This body of evidence led us to hypothesize that CNS EC-ICOSL could be involved in mediating immune cell adhesion to the BBB. To prove this, we performed T cell migration assays as well as static T cell adhesion assays on anti-ICOSL-treated pMBMECs. We found significantly reduced numbers of adhering MOG_35–55_-specific Th1 and Th17 cells under static conditions when ICOSL was blocked on IL-1β-stimulated pMBMECs (Figure 7, A–C). We next assessed the postarrest behavior of Th1 cells under physiological flow conditions. The numbers of arrested T cells showed a trend toward lower numbers of cells arresting when ICOSL was blocked; however, due to the variance across the individual experiments, this trend did not reach statistical significance (Figure 7D). The postarrest behavior, which we categorized into fractions of T cells that were detaching, probing, probing followed by diapedesis, crawling, and crawling followed by diapedesis, was unaltered when ICOSL was blocked (Figure 7E). Also, the crawling distance and crawling speed of the T cells that successfully performed diapedesis following crawling on the pMBMECs was not changed (Figure 7, F and G). Taken together, ICOSL promoted the arrest of Th1 and Th17 cells to the monolayer without influencing the postarrest behavior of T cells on pMBMECs under physiological flow.

## Discussion

In this study, we identified a previously unknown role of CNS-EC A20 in neuroinflammation. Our results show that the loss of A20 in CNS-ECs strongly increased the risk of developing EAE upon transfer of encephalitogenic T cells into naive recipients and accelerated disease onset. ECs lacking A20 were dramatically activated, which resulted in the upregulation of cell adhesion molecules like ICAM-1 and VCAM-1, but also ICOSL. We propose here that A20 serves as a gatekeeper for the activation of ECs, and its absence drives transmigration of encephalitogenic T cells across the BBB, and, by this, at least partially, contributes to the increased disease susceptibility.

Supporting evidence for this hypothesis comes from the observation that innate immune activation, for example mediated by CFA used in the active EAE induction protocol, drives endothelial activation and promotes upregulation of adhesion molecules already before onset of disease, suggesting a causal role for endothelial activation in disease initiation (13, 55, 56). Interestingly, and in contrast to A20-deficiency in microglia and astrocytes (28, 29), we did not observe alterations in an actively induced EAE disease in mice with A20-deficient ECs. It seems likely that the endothelial activation in response to CFA in the active EAE model masks the effects driven by A20 deletion in CNS-ECs and could thus explain the difference seen in AT-EAE but not active EAE outcomes.

We observed an increased infiltration of immune cells into the CNS of A20^ΔCNS-EC^ mice in steady state and after AT-EAE. To enter the CNS, immune cells need to cross the BBB, which represents a key step in the pathology of CNS autoimmune diseases such as MS. Both immune cells and BMECs require a certain state of activation to allow immune cells to cross the BBB. The influence of cytokines and other inflammatory mediators secreted by activated T cells but also innate immune cells on the endothelium has been extensively described (57–59). In particular, proinflammatory cytokines like TNF or IL-1β that activate the NF-κB pathway are essential in mediating the upregulation of cell adhesion molecules on BMECs that promote the infiltration of pathogenic T cells and the development of CNS autoimmunity (13, 60, 61). Upregulation of ICAM-1 and VCAM-1 in CNS-ECs has been described in A20^–/–^ mice in the steady state; however, it was suggested that this upregulation of adhesion molecules was due to the overall heightened cerebral inflammation present in A20^–/–^ mice (27). In opposition to this, we show here that ICAM-1 and VCAM-1 upregulation in steady-state CNS-ECs was driven by an endothelial-intrinsic response to A20-deficiency, as A20^ΔCNS-EC^ mice manifested a strong expression of both molecules without external inflammatory stimulation.

Our results further indicate that rather excessive adhesion molecule expression and not BBB breakdown are responsible for the pronounced CNS immune cell infiltration in A20^ΔCNS-EC^ mice. It was earlier proposed that NF-κB signaling in BMECs is essential for maintaining barrier integrity (22). Disruption of NF-κB signaling through CNS EC-specific deletion of NEMO or TAK1 resulted in an increased BBB permeability, which was suggested to result from impaired A20-mediated occludin stabilization (22). Although we could confirm that A20-deficiency in CNS-ECs led to decreased occludin levels, this reduction did not cause BBB or BSCB breakdown in our study, underscoring the debate about the necessity of occludin for barrier integrity (62–65). Our data further corroborates earlier reports of A20^–/–^ and A20^+/–^ mice, which also did not manifest any signs of BBB disruption (27, 66). We can thus conclude that activation of BMECs through loss of A20 provokes expression of cell adhesion molecules, which promotes the firm adhesion of immune cells to the BBB and facilitates their entry into the CNS.

Supporting our findings, we further found a more severe disease pathology in the MCAO model, a model involving sterile CNS inflammation, in A20^ΔCNS-EC^ mice. Interestingly, T cells infiltrate into the injured areas as early as 24 hours after MCAO induction (67), and blocking of the α4-integrin unit of VLA-4, the binding partner of VCAM-1, reduces the infarct volumes by restricting CNS T cell infiltration (68, 69). It is thus possible that the increased infarct areas in A20^ΔCNS-EC^ arise from a more pronounced T cell infiltration due to the massive EC activation.

The success of targeting the VLA-4 subunit α4-integrin with natalizumab in MS therapy points out the attractiveness of interfering with transmigration of immune cells across the BBB in diseases involving CNS inflammation. Yet, due to the potential side effects of this treatment strategy it is necessary to identify alternative targets for therapeutic intervention. In recent years, alternative cell adhesion molecules involved in immune cell transmigration across the BBB have been identified. ALCAM was found to be an important adhesion molecule guiding B cell and monocyte trafficking across the BBB, yet deletion of this molecule dramatically influences barrier integrity and causes more severe EAE disease (48, 49, 70). Also, MCAM was implicated in several studies to be essential for CD8^+^ as well as Th17 cell migration across the BBB (50, 51, 71). More recently, DICAM was also found to be involved in Th17 cell migration across the BBB and was suggested as a potential target in progressive MS forms (52). While progress has been made in recent years, the spectrum of molecular interactions occurring at the CNS-peripheral interface is certainly not yet disentangled in its entirety. We here identified A20-regulated ICOSL as an adhesion molecule contributing to the firm adhesion of MOG_35–55_ specific proinflammatory Th1 and Th17 cells to the BBB. Although the reduction in T cell adhesion when ICOSL was blocked appeared rather mild, the effect is in line with studies investigating T cell adhesion to other adhesion molecules (52, 72). ICOSL was only recently reported to mediate adhesion of podocytes by binding to the α_V_β_3_ integrin through its RGD motif (54). Interestingly, Roussel et al. earlier introduced a human mutation in ICOSL, which was found in a patient with a combined immunodeficiency syndrome (73). The authors identified an involvement of endothelial ICOSL in mediating the transmigration of T cells and neutrophils across an in vitro endothelial barrier (73). Here, we corroborate these findings and extent the involvement of endothelial ICOSL in immune cell adhesion from the periphery to brain-derived pMBMECs. Future studies are, however, necessary to determine the exact binding partner of ICOSL that mediates this adhesion and to further characterize the spectrum of immune cells having the capacity to bind to ICOSL.

We could further underscore the functional role of CNS EC-ICOSL in the development of autoimmune neuroinflammation by in vivo silencing of ICOSL with a CNS-microvascular endothelial specific AAV. Although the GFP signal from our AAV-BR1-shIcosl likely underrepresents the percentage of transduced cells due to the comparably weak RSV promoter driving GFP expression, we could confirm the efficiency of our construct to knock down ICOSL specifically in CNS-ECs, both in vitro and in vivo. More importantly, we were able to partly rescue the accelerated disease outcome in A20^ΔCNS-EC^ mice by treating them with AAV-BR1-shIcosl, although not reaching statistical significance. Notably, and opposed to observations from Roussel et al., protein levels of ICAM-1 and VCAM-1 molecules were not affected by silencing of ICOSL (73). The high levels of these molecules remaining in A20^ΔCNS-EC^ mice might explain the rather mild amelioration in the day of disease onset and the overall disease severity upon treatment with AAV-BR1-shIcosl. Nevertheless, silencing of ICOSL on CNS-ECs restricted the infiltration of transferred encephalitogenic T cells into the CNS of A20^ΔCNS-EC^ mice, confirming its contribution to T cell mediated autoimmunity.

In agreement with these observations, our results additionally showed that the day of active EAE onset could be delayed when ICOSL was knocked down in CNS-ECs of WT mice. As transmigration of immune cells through the BBB precedes the development of EAE symptoms (74, 75), our data suggest that the delayed disease development is due to an impairment of T cell adhesion to the BBB. The phenotype we observed is in accordance with EAE data from mice in which other cell adhesion molecules (ICAM-1, ICAM-2, VCAM-1) were targeted (36, 76). Targeting of CNS microvascular EC-ICOSL thus seems to be an attractive approach for controlling infiltration of pathogenic immune cells in the context of autoimmune neuroinflammation. However, ICOSL is well-known to also function as costimulatory molecule in an immunological context (77–79) and earlier studies targeting ICOS indicated a protective effect of ICOS-ICOSL interaction in the priming phase of EAE (80, 81). Thus, anti-ICOSL treatment for CNS autoimmunity needs to be carefully evaluated, as the desired effect strongly relies on cell specificity as well as the time point of therapy.

In conclusion, our study sheds light on two important aspects in the pathology of CNS inflammation. Firstly, our data demonstrate a protective role for CNS endothelial A20 by preventing the excessive expression of cell adhesion molecules and thus regulating the infiltration of immune cells into the CNS. Secondly, we discovered ICOSL as an A20-regulated cell adhesion molecule mediating the firm adhesion of Th1 and Th17 cells to the BBB. Overall, this study suggests ICOSL as a potential therapeutic target in the treatment of CNS autoimmune diseases such as MS.

## Methods

### Mice.

All mice used were on the C57BL/6J background and housed under specific pathogen-free conditions. A20^fl/fl^ mice (82) were crossed to Slco1c1-CreER^T2^ mice (32) (A20^ΔCNS-EC^) and used for A20 Western blot analysis and immunofluorescent stainings of brain vessels with collagen IV, CD31, occludin and VCAM-1, and MCAO and active EAE in animal facility 2. For all other experiments analyzing A20^ΔCNS-EC^ mice, A20^fl/fl^ mice (42) were crossed with Slco1c1-CreER^T2^ mice (32). For generation of A20^ΔEC^ mice, A20^fl/fl^ mice (42) were crossed with Cdh5(PAC)-CreER^T2^ (33) mice. Cre-negative A20^fl/fl^ littermates were used as controls. For sequencing experiments, A20^ΔCNS-EC^ mice were crossed with R26R-EYFP reporter mice (83). For AT-EAE experiments, Ly5.1 mice (B6.SJL-PtprcaPepcb/BoyCrl) were used as donor mice.

### TAM treatment.

A 20 mg/mL TAM (Sigma-Aldrich) solution was prepared by suspension in olive oil (Sigma-Aldrich) containing 5 % ethanol. TAM was dissolved by rotation overnight at 4°C. Mice were injected i.p. with 100 μL (2 mg TAM) at 5 (for Slco1c1-CreER^T2^ strains) or 3 (for Cdh5(PAC)-CreER^T2^ strains) consecutive days at the age of 6–7 weeks and mice were used in experiments 2–4 weeks thereafter, unless otherwise indicated. A20^fl/fl^ littermate controls were treated equally.

### Active EAE induction.

An emulsion of MOG_35–55_ in CFA was prepared by mixing 1 mg/mL MOG_35–55_ (GenScript) in PBS with CFA (BD Biosciences) supplemented with *Mycobacterium tuberculosis* H37RA (BD Biosciences). 50 μg MOG_35–55_/CFA was injected subcutaneously into the tail base. At the day of immunization and 2 days later, 150 ng pertussis toxin (PTx, List Biological Laboratories) in PBS was applied i.p. unless otherwise stated. For active EAE induction in facility 2 (Supplemental Figure 1, E–H), mice were immunized with an immunizing emulsion (EK-2110, Hooke Laboratories) containing a MOG_35–55_ peptide in CFA as described before (84). Additionally, mice were treated with 400 ng PTx i.p. on the day of immunization and 1 day later.

Mice were weighed daily and clinical scores were documented as follows: 0, no disease; 0.5: limp tail; 1: paralyzed tail; 1.5: weakened righting reflex; 2: no righting reflex; 3: partial paralysis of hind legs; 3.5: paralysis of 1 hind leg; 4: paralysis of both hind legs.

### AT EAE induction.

Ly5.1 mice were immunized with MOG/CFA and PTx as described above. Ten days after immunization, spleen, inguinal, and paraaortic lymph nodes were harvested and single-cell suspensions were prepared and cultured in vitro in the presence of MOG_35–55_ peptide (20 μg/mL), anti-IFNγ (10 μg/mL; BioXCell), and IL-23 (15 ng/mL; Miltenyi Biotec). After 4 days, cells were harvested and examined for blasting lymphocytes, as based on forward and side scatter properties in flow cytometry. Cell suspensions were adjusted to 50 × 10^6^ blasting cells/mL, and 100 μL of this suspension was injected i.v. into the tail veins of recipient mice, accompanied by i.p. injections of PTx (150 ng) the same day and 2 days later. Mice were weighed daily and the clinical scores were assessed according to the scoring system described above.

### Permanent MCAO.

The ischemic stroke model was performed as described before (85). Briefly, mice were anesthetized using tribromoethanol (2.5%, 15μL/g body weight, i.p.). After a skin incision and removal of the left temporal muscle a burr hole was drilled, through which the stem of the middle cerebral artery was occluded by electrocoagulation (Model ICC50, Erbe). After suturing, mice were placed under a heating lamp until they fully recovered. After 48 hours, mice were perfused intracardially under deep anesthesia and brains were removed. Coronal cryosections were cut every 400 μm and stained using a silver technique (86). Infarcted areas were measured using ImageJ and infarct volume, edema, and edema-corrected infarct volume were calculated as described before (86).

### Immunofluorescence staining.

Immunofluorescence stainings were performed on fixed frozen sections of 10–20 μm. Primary antibodies against CD3ε (145-2C11, Armenian hamster monoclonal, Thermo Fisher Scientific, 1:100), NeuN (1B7, Mouse monoclonal, Thermo Fisher Scientific, 1:500), CD31 (rabbit polyclonal, abcam, 1:100 or MEC 13.3 rat monoclonal, BD Pharmingen, 1:500), laminin 1+2 (ab7463, rabbit polyclonal, abcam, 1:500), collagen IV (ab6586, rabbit polyclonal, abcam, 1:1,000), occludin (13409-1-AP, rabbit polyclonal, Proteintech, 1:1,000), and VCAM-1 (MVCAM.A, rat monoclonal, BD Pharmingen, 1:1,000) were incubated over night at 4°C. Sections were washed and secondary antibodies against rabbit IgG (CF488A, Sigma-Aldrich, 1:800, CF 555, Sigma-Aldrich, 1:800, or Cy3, Jackson ImmunoResearch Labs, 1:400), Armenian hamster (127-605-160, Alexa Fluor 647, Jackson ImmunoResearch Labs, 1:800), mouse IgG (CF 647, Sigma-Aldrich, 1:500) or rat IgG (Alexa 488, A-21208, Thermo Fisher Scientific, 1:400) were added for 45 minutes to 1.5 hours at room temperature. Afterward, sections were mounted with DAPI Fluoromount-G Mounting Medium (SouthernBiotech) or Mowiol 4-88 (Carl Roth) and images were acquired at a TCS SP8 inverse confocal microscope (Leica) or a DMI 6000B fluorescence microscope (Leica). Images were analyzed with ImageJ software as described before (22).

### Culture of pMBMECs.

Cortices from 8–12-week-old WT mice were used for pMBMEC isolation, as described previously (87). Cells were seeded on 48-well culture dishes coated with Matrigel (ECM Gel from Engelbreth-Holm-Swarm murine sarcoma, supplied by Sigma-Aldrich). Culture medium was supplemented with 20% FCS, 2% nonessential amino acids, and 5 μg/mL gentamicin. Additionally, 1 ng/mL human fibroblast growth factor (FGF) (Sigma-Aldrich) and 4 μg/ml puromycin (Gibco) were added for the first 48 hours of culture. Afterward, the medium was changed to puromycin-free medium supplemented with FGF and was changed every other day.

### Leukocyte cell isolation from CNS for flow cytometry.

CNS tissue was dissected from mice transcardially perfused with 0.9% NaCl solution (Sigma-Aldrich) and digested with 2 mg/mL collagenase II (Gibco) and 25 μg/mL DNase I (Roche) for 20 minutes at 37 °C and subsequently homogenized with a 18-G needle. Cells were then separated using a 70%–37%–30% Percoll (Sigma-Aldrich) gradient centrifugation for 40 minutes at 500*g* and 16 °C. Cells at the 70%/37% interphase were carefully collected and washed in PBS/FCS before 10 minutes centrifugation at 500*g*. To characterize MOG-specific T cells and cytokine production, cells were plated in 96-well U-bottom plates and reactivated with 20 μg/mL MOG_35–55_ peptide in the presence of brefeldin A (Sigma-Aldrich) in T cell medium for 6 hours at 37 °C. Afterward, cells were harvested and stained for flow cytometry analysis.

### Endothelial cell isolation for flow cytometry.

For CNS EC-isolation, mice were sacrificed and transcardially perfused as described above. The dissected CNS tissue was digested with 2 mg/mL papain (Sigma-Aldrich) solution containing 25 μg/mL DNase I (Roche) for 30 minutes at 37 °C. During incubation, tissue was mechanically homogenized using the gentleMACS Dissociator (Miltenyi Biotec). The resulting cell suspension was filtered through a 70 μm cell strainer and centrifuged with a 22 % Percoll gradient for 30 minutes at 300*g* and 15 °C. The pellet was used for flow cytometry staining.

### Flow cytometry analysis.

Before antibody staining, Fc receptors were blocked for 20 minutes using Fc-block (5 μg/mL) (BioXCell). Single cell suspensions were stained for 30 minutes at 4 °C with antibodies against CD4 PerCP (GK1.5, rat monoclonal, 1:500, BioLegend), CD45 BV510 (30-F11, rat monoclonal, 1:200, BioLegend), CD45.1 FITC (A20, mouse monoclonal, 1:1,000, BioLegend), CD11b PECy7 (M1/70, rat monoclonal, 1:1,000, eBioscience), Ly6C V450 (AL-21, rat monoclonal, 1:300, BD Bioscience), Ly6C PerCP (HK1.4, rat monoclonal, 1:100, BioLegend), Ly6G PE (1A8, rat monoclonal, 1:1,000, BioLegend), CD11c APC (HL3, rat monoclonal, 1:800, BD Bioscience), CD19 PerCP (6D5, rat monoclonal, 1:400, BioLegend), TCRβ APC (H57-597, rat monoclonal, 1:1,000, BioLegend), CD31 PE (MEC 13.3, rat monoclonal, 1:100, BioLegend), CD31 PerCP-Cy5.5 (390, rat monoclonal, 1:100, BioLegend), ICAM-1 APC (YN1/1.7.4, rat monoclonal, 1:300, BioLegend), VCAM-1 PE-Cy7 (429 [MVCAM.A], rat monoclonal, 1:500, BioLegend), ICOSL (CD275), and PE (HK5.3, rat monoclonal, 1:100, BioLegend).

Afterward, where indicated, cells were fixed and permeabilized with Cytofix/Cytoperm (BD Bioscience) or Foxp3/Transcription Factor Staining Buffer Set (eBioscience) and stained overnight at 4 °C with intracellular antibodies. To specifically gate on MOG-specific cells, cells were stained with anti-CD154 (CD40L) APC (MR1, hamster monoclonal, 1:200, BioLegend), and cytokine production was assessed by staining with IL-17A eFl450 (eBio17B7, rat monoclonal, 1:300, eBioscience), IFN-y PE-Cy7 (XMG1.2, rat monoclonal, 1:1,000, eBioscience), and GM-CSF PE (MP1-22E9, rat monoclonal, 1:200, eBioscience). Staining for Foxp3 was performed with Foxp3-FITC (FJK-16s, rat monoclonal, 1:200, eBioscience).

Cells were acquired with a FACSCanto II cytometer (BD Bioscience) using FACS Diva software (BD Bioscience). Flow cytometry data was analyzed with FlowJo software version 10 (BD Bioscience). For all analyses, doublets (FSC and SSC properties) and dead cells (dye inclusion, fixable viability dye APC-ef780 eBioscience) were excluded.

### FACS of CNS-ECs.

Endothelial cells from the spinal cord were isolated and subsequently stained with a surface marker antibody panel and directly acquired at a FACSAria III (BD Bioscience) with a 100 μm nozzle.

### RNA isolation and RNA-Seq.

For RNA-Seq, 500 cells were sorted into lysis buffer and full-length cDNA was synthesized using the SMART-seq^â^ v4 Ultra^â^ Low Input Kit (Takarabio). A total of 1 ng of the resulting cDNA was used for library preparation (Illumina Nextera XT DNA Library Prep Kit) according to the manufacturer’s instructions. Quantity was assessed using Invitrogen’s Qubit HS assay kit, and library size was determined using Agilent’s 2100 Bioanalyzer HS DNA assay. Barcoded RNA-Seq libraries were onboard clustered using HiSeq Rapid SR Cluster Kit v2 using 8pM and 59bps and were sequenced on the Illumina HiSeq2500 using HiSeq Rapid SBS Kit v2 (59 Cycle). The raw output data of the HiSeq was preprocessed according to the Illumina standard protocol.

### RNA-Seq data analysis.

Quality control on the sequencing data was performed with the FastQC tool (version 0.11.8, https://www.bioinformatics.babraham.ac.uk/projects/fastqc/). RNA-Seq reads were aligned to the ENSEMBL Mus_musculus.GRCm38 reference genome. The corresponding annotation (ENSEMBL v76) was retrieved from ENSEMBL FTP website. The STAR aligner (version 2.6.1a) was used to perform mapping to the reference genome. Alignments were processed with the featureCounts function of the Rsubread package (version 2.10.0), using the annotation file also used for supporting the alignment. The exploration, modeling, and interpretation of the expression data followed the protocols defined by Ludt et. al (2022) (88). Exploratory data analysis was performed with the pcaExplorer package (version 2.22.0). Differential expression analysis was performed with DESeq2 package (version 1.36.0), setting the FDR cutoff to 0.05. Accurate estimation of the effect sizes (described as log_2_ fold change) was performed using the apeglm shrinkage estimator (version 1.18.0). Gene expression profiles were plotted as heatmaps (color-coded standardized z-scores for the expression values, after regularized logarithm transformation) to enable comparison across samples, created with the GeneTonic package (version 2.0.3) (89). For functional annotation of Gene Ontology (GO) and analysis of KEGG pathway enrichment, the web-based DAVID 2021 tool was used.

### FITC-dextran permeability assay.

FITC-dextran (avg. mol. weight 3,000-5,000 Da, Sigma-Aldrich) was dissolved at 2 mM in PBS. 100 μL of this stock solution was injected i.p. into the mice. After 15 minutes, mice were sacrificed and perfused with 0.9% NaCl solution. Spinal cords and half brains were weighed and subsequently homogenized in 200 μL (spinal cord) or 500 μL (half brain) PBS in metal bead lysing matrix tubes (MP Biomedicals) using the FastPrep-24 (MP Biomedicals) system. Tubes were centrifuged at 15,000*g* for 20 minutes at 4°C, and 100 μl of the supernatant were transferred into a 96 well flat black plate (Greiner). FITC fluorescence was measured with a Spark plate reader (Tecan) with an excitation of 490/10 nm and an emission of 520/10 nm and raw fluorescence units (RFU) were normalized to tissue weights.

### RNA isolation from pMBMECs and real time (RT) PCR.

Isolation of RNA from cultured pMBMECs was performed with the ReliaPrep RNA Cell Miniprep System (Promega) following manufacturers guidelines. RNA concentrations were determined by measuring absorbance using the NanoQuant Plate (Tecan) at an Infinite M200 pro plate reader (Tecan). cDNA was synthesized using 200–1000 ng of total RNA with the QuantiTect^â^ Reverse Transcription Kit (Qiagen) and subsequently used for qPCR, which was performed with the StepOnePlus RT PCR System (Life Technologies) using SYBR Green reagent (Promega). Fold enrichment was calculated using the ^ΔΔ^CT method normalized to hypoxanthin-guanin-phosphoribosyltransferase (*Hprt*) as house-keeping reference. *Tnfaip3* primer were ordered as QuantiTect Primer Assay (QT00134064, Qiagen). *Icosl* primer (forward: 5′- AGGCTCCCTTGGACATCTCG -3′; reverse: 5′- CAGTACAGCAAGGACGGGGA -3′) were self-designed using primer-BLAST tool from the NCBI and were synthesized by Metabion.

### Western blot.

Western blotting for the A20 protein was performed on primary brain endothelial cells of A20^fl/fl^ and A20^ΔCNS-EC^ mice. After reaching confluence, cells were lysed and Western blotting was performed as described before (22) using antibodies targeting A20 (A-12, mouse monoclonal, Santa Cruz, 1:500) and actin (C-11, goat polyclonal, Santa Cruz, 1:1,000) and secondary antibodies against mouse IgG (HRP-coupled, Santa Cruz, 1:1,000) and goat IgG (HRP-coupled, DAKO, 1:10,000), respectively.

### In vitro T cell static adhesion assay.

pMBMECs were isolated and cultured as described above for 7 days on μ-dishes (ibidi GmbH). After 4 days, cells were stimulated with 20 ng/mL IL-1β for 72 hours. Th1 or Th17 cells from 2D2 C57BL/6J mice were thawed and rested for approximately 4 hours in T cell medium and subsequently labelled with 1 μM CellTracker Green (Thermo Fisher Scientific) for 30 minutes. pMBMECs were incubated with 15 μg/mL anti-mouse ICOSL (HK5.3, rat IgG2a monoclonal, BioXCell) or 15 μg/mL negative control (MJ7/18, rat IgG2a anti mouse endoglin (76)) for 30 minutes. 100,000 T cells were added to the pMBMECs and were allowed to adhere for 30 minutes on a shaking platform. Wells were washed with PBS and fixed with 1% PFA for 10 minutes before mounting on glass slides with DAPI. Images were acquired at an Eclipse E600 microscope (Nikon) and adhering T cells were counted using ImageJ software (National Institute of Health).

### In vitro T cell migration assay under physiological flow.

In vitro live-cell imaging of T cell diapedesis across pMBMECs was performed as described before (90). In brief, pMBMECs were isolated and cultured for 7 days on μ-dishes (ibidi GmbH). After 4 days, cells were stimulated with 20 ng/mL IL-1β for 72 hours. 2D2 Th1 cells were resuspended at 1 × 10^6^cells/mL in T cell medium. Prior to the experiment, pMBMECs were treated with 15 μg/mL anti-mouse ICOSL (HK5.3, rat IgG2a monoclonal, BioXCell) or 15 μg/mL negative control (MJ7/18, rat IgG2a anti mouse endoglin (76)) for 30 minutes at 37°C. Accumulation of 2D2 Th1 on pMBMECs in the flow chamber was allowed for 4 minutes at a low shear (0.1 dyn/cm^2^), followed by physiological shear (1.5 dyn/cm^2^) for an additional 20 minutes, for a total recording time of 24 minutes. Image acquisition was performed at ×10 magnification with the AxioObserver inverted microscope (Carl Zeiss) with differential interference contrast using the camera Evolve Delta (Teledyne Photometrics). Image frames were recorded every 10 seconds. Image analysis was performed using ImageJ software (National Institute of Health). T cell postarrest behavior was defined and expressed as described previously (91). T cell crawling tracks were evaluated after manual tracking of individual T cells using the manual tracking plug-in of ImageJ. Distance and speed of crawling tracks were evaluated using chemotaxis and migration tool (version 2.0, Ibidi GmbH).

### AAV design and applications.

For knockdown of ICOSL specifically in CNS-ECs AAV-BR1 (45) was used to deliver a short hairpin (sh) RNA against *Icosl* carrying the sense sequence GCAGAAAGTTTCACTGGAAATCTC to the target cells. The AAV-BR1-RSV-GFP-H1-shRNA_Icosl and the control AAV-BR1-RSV-GFP-H1-shRNA_scrambled constructs were produced by the Viral Vector Production Unit of the Universitat Autònoma Barcelona, Spain. The AAVs were delivered to us ready for use.

For in vitro experiments, pMBMECs grown in 48-well plates were transfected with 0.6 × 10^6^ genomic particles (gp) per well 4 days after seeding. For in vivo experiments, constructs were delivered i.v. at a concentration of 1.8 × 10^11^ gp in 100 μL sterile PBS. AAV-BR1-eGFP was used as described elsewhere (45).

### Statistics.

Statistical analyses were performed with Prism v9 software (GraphPad). Symptom-free survival data are presented as Kaplan-Meier survival curves, and differences between groups were analyzed by log-rank test. Differences between 2 groups were tested using a 2-tailed unpaired Student’s *t* Test. When more than 2 groups were compared, a ordinary 1-way ANOVA with Tukey’s or Dunnett’s multiple comparisons test was used. Comparisons in more than one category were performed with a 2-way ANOVA with Šidák’s multiple comparisons test. All values are represented as mean ± SEM unless otherwise stated. *P* values were considered significant with p<0.05.

### Study approval.

All animal experiments were approved by the local administrations (Landesuntersuchungsamt Koblenz, Germany; Ministerium für Energiewende, Landwirtschaft, Umwelt und ländliche Räume, Kiel, Germany; individual approval numbers G20-1-049 and G22-1-005). Experiments were performed in accordance with the guidelines from the translational animal research center (TARC) Mainz, Germany. All efforts were made to minimize suffering of the mice.

### Data availability.

The RNA-Seq data presented in this manuscript have been deposited in the Gene Expression Omnibus (GEO) under accession number GSE221318. All other data is available in the Supporting Data Values file.

## Author contributions

LJ performed experiments, analyzed data, prepared figures, and wrote the manuscript. SS, KM, JL, MAK, JCA, and JW performed experiments and analyzed data; FM performed bioinformatic analysis; NR, IP, and CS helped with experiments, data analysis, and discussion. ES helped with experiments and manuscript writing. MK and TB provided reagents and guided preparation of samples for RNA-Seq. KK helped with histology and confocal imaging. MHHS, DS, and GVL provided mouse strains. JK provided the AAV-BR1 plasmid. BE and MS were associated with conceptualization and supervision of the project, funding acquisition, and finalization of the manuscript. AW supervised the project, acquired funding and wrote the manuscript together with LJ.

## Supplementary Material

Supplemental data

Supporting data values

## Version 1 10/19/2023

In-Press Preview

## Version 2 12/15/2023

Electronic publication
