# Supplementary material for: A20 regulates lymphocyte adhesion in murine neuroinflammation by restricting endothelial ICOSL expression in the CNS
Source: J Clin Invest. 2023 Dec 15;133(24):e168314. doi: 10.1172/JCI168314 (PMC10721159; doi:10.1172/JCI168314)
Supplement: Supplemental data [file jci-133-168314-s037.pdf]

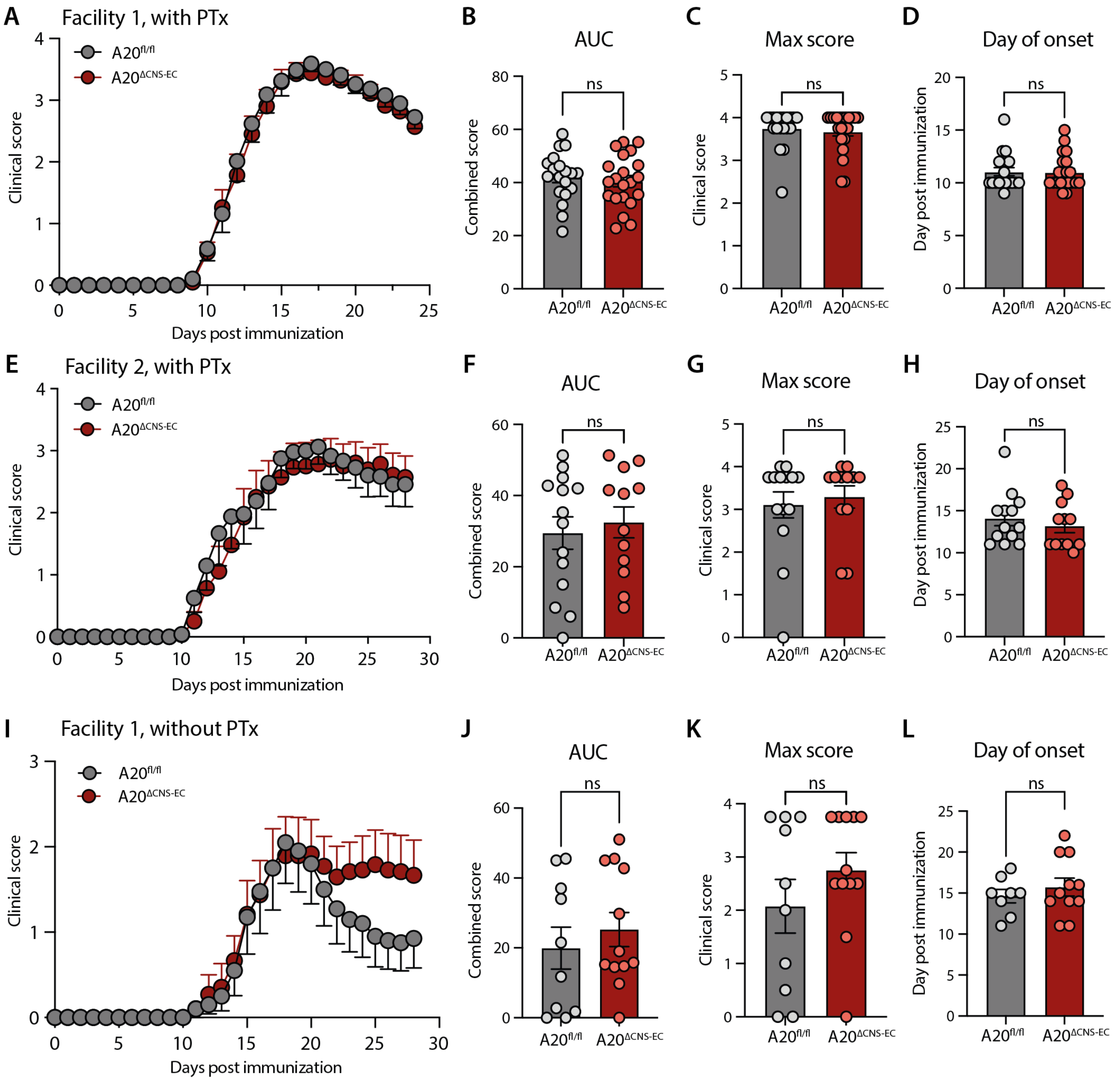

Supp. Figure 1: Loss of CNS EC-A20 does not alter active EAE disease

(A-H) A20$^{\Delta CNS\text{-}EC}$ and littermate control mice were immunized with $MOG_{35\text{-}55}$ in CFA and pertussis toxin two to four weeks after tamoxifen treatment in two different animal facilities. Clinical signs of EAE were monitored daily. (A) Clinical signs of EAE are shown as mean clinical disease scores +/- SEM. (B) Area under the curve (AUC), (C) maximum disease score and (D) day of disease onset analyses of clinical course shown in (A). Data is pooled from two independent experiments with n = 19-21 mice per group. (E) Clinical signs of EAE are shown as mean clinical disease scores +/- SEM. (F) AUC, (G) maximum disease score and (H) day of disease onset analyses of clinical course shown in (E) (n = 12-14 per group). (I-L) A20$^{\Delta CNS\text{-}EC}$ and littermate control mice were immunized with $MOG_{35\text{-}55}$ in CFA four weeks after tamoxifen treatment in facility 1. Clinical signs of EAE were monitored daily. (I) Clinical signs of EAE are shown as mean clinical disease scores +/- SEM. (J) AUC, (K) maximum disease score and (L) day of disease onset analyses of clinical course shown in (I). Data is pooled from two independent experiments with n = 10-12 mice per group. Statistical significance was determined by two-tailed unpaired Student's t-test. ns = not significant.

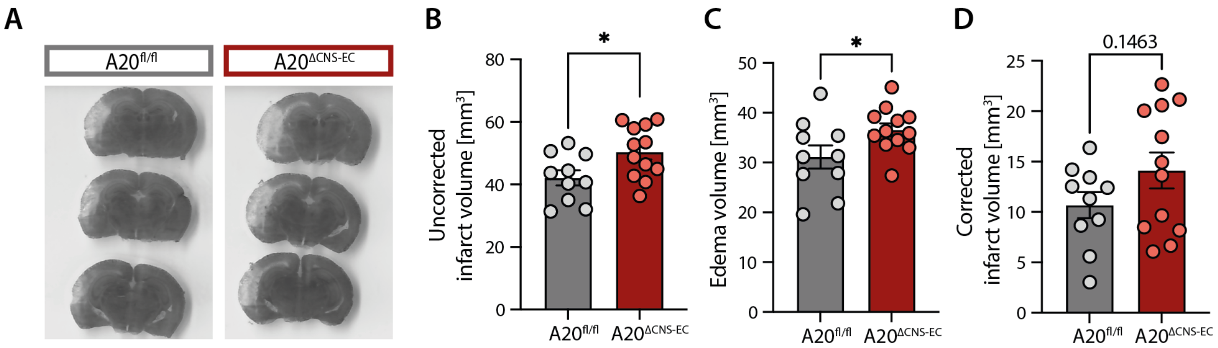

Supp. Figure 2: Loss of CNS EC-A20 mildly accelerates MCAO pathology

A20$^{\Delta CNS\text{-}EC}$ and littermate control mice were exposed to middle-cerebral artery occlusion (MCAO) two weeks after tamoxifen treatment (n = 10-12 mice per group). (A-D) Coronal cryosections were cut every 400 µm and stained using a silver technique. (A) Exemplary images of silver-stained cryosections. (B) Uncorrected infarct volumes, (C) Edema volumes and (D) edema-corrected infarct volumes were quantified. Statistical significance was determined by two-tailed unpaired Student's t-test (B-D). * $p<0.05$

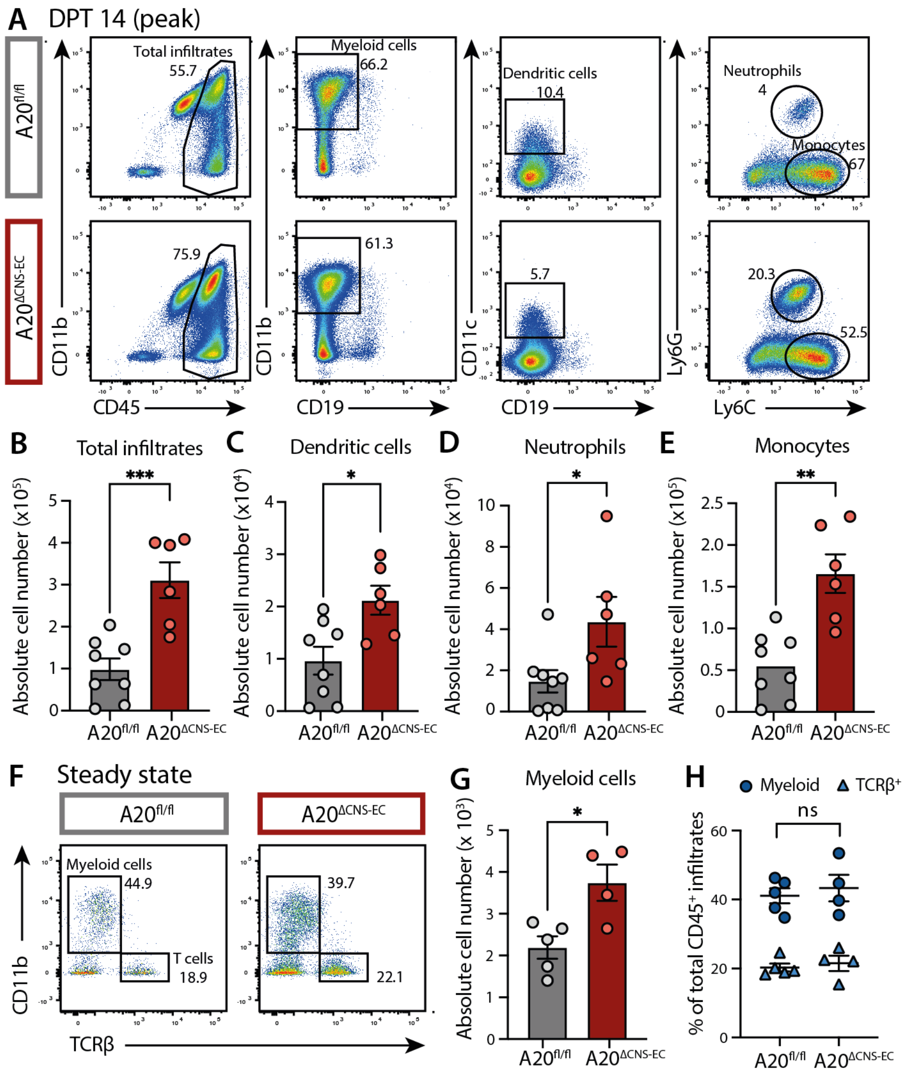

Supp. Figure 3: Loss of CNS EC-A20 drives myeloid cell infiltration during EAE and in the steady state

(A-E) AT-EAE disease was induced in A20$^{\Delta CNS-EC}$ mice and A20$^{fl/fl}$ littermate controls. At the peak of the disease (DPT14) SC-infiltrating myeloid cells were isolated and analyzed by flow cytometry. Data is representative for 2 individual experiments with n = 6-8 mice per group. (A) Representative gating strategy of myeloid subpopulations in A20$^{\Delta CNS-EC}$ mice and A20$^{fl/fl}$ controls. Total infiltrates were gated as CD45$^+$ cells from single, live cells. Amongst them, myeloid cells were determined as CD19$^-$ CD11b$^+$ cells. From this population, dendritic cells were identified as CD11c$^+$ cells, neutrophils as Ly6G$^+$ Ly6C^{int} and monocytes as Ly6G$^-$ Ly6C$^+$. (B) Absolute cell numbers of total CD45$^+$ infiltrates, (C) dendritic cells, (D) neutrophils and (E) monocytes. (F-H) Flow cytometry of CNS immune cells in the steady state of A20$^{\Delta CNS-EC}$ and A20$^{fl/fl}$ mice one week after TAM treatment. Cells were isolated from pooled spinal cord and brain tissue. Data is representative for 3 individual experiments with n = 4-5 mice per group. (F) Representative gating strategy for CD11b$^+$ myeloid and TCRβ$^+$ T cells; pre-gated as single, live CD45$^+$ cells. (G) Absolute cell numbers of CD11b$^+$ myeloid cells. (H) Frequencies of TCRβ$^+$ T cells and CD11b$^+$ myeloid cells amongst all CD45$^+$ infiltrating cells. Statistical significance was determined by two-tailed unpaired Student's t-test (B-E, G) or two-way ANOVA (H). ns = not significant, * $p<0.05$, ** $p<0.01$, *** $p<0.001$.

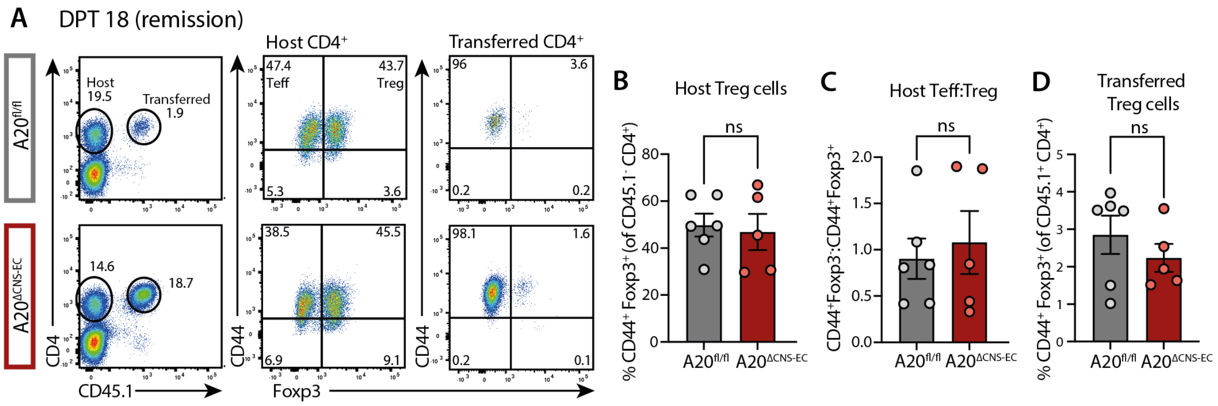

Supp. Figure 4: A20-deficiency in CNS-ECs does not cause alterations in CNS Treg cell frequencies
(A-D) AT-EAE disease was induced in A20$^{\Delta CNS\text{-}EC}$ mice and A20$^{fl/fl}$ littermate controls. In the remission phase (DPT18) spinal cord-infiltrating Treg cells were analyzed by flow cytometry (n = 5-6 mice per group). (A) Representative gating strategy for host-derived (CD45.1$^-$) and transferred (CD45.1$^+$) CD4$^+$ T cells and amongst them CD44$^+$ versus Foxp3$^+$ cells. (B) Frequency of host Treg cells, (C) ratio of host effector T cell to regulatory T cell (Teff:Treg) ratio, and (D) frequency of transferred Treg cells were quantified. Statistical significance was determined by two-tailed unpaired Student's t-test. ns = not significant.

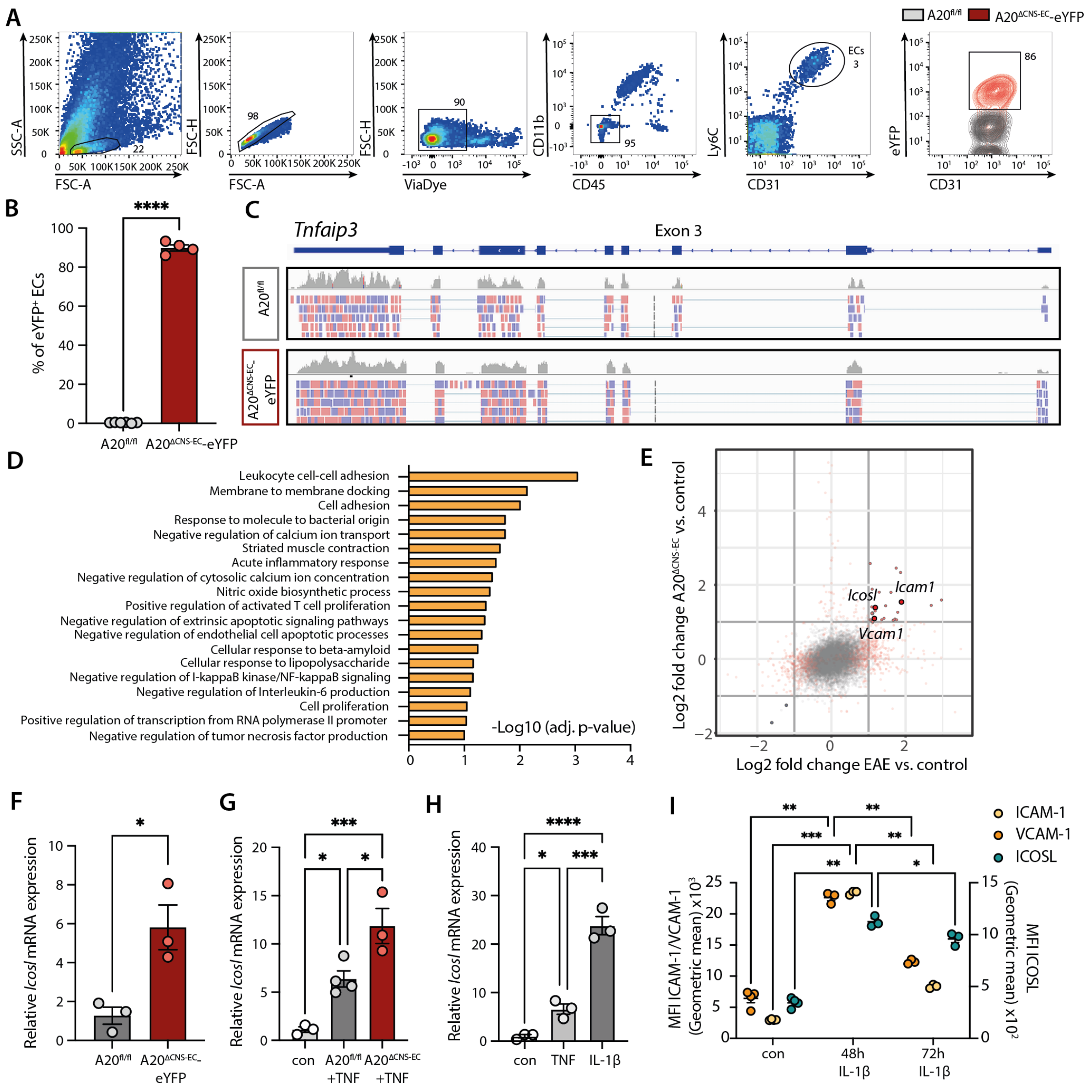

Supp. Figure 5: Icosl is upregulated in A20-deficient endothelial cells and behaves similar to other adhesion molecules

(A) Representative gating strategy for sorting of ECs from SC of naïve A20$^{\Delta CNS\text{-}EC}$-eYFP and A20$^{fl/fl}$ mice. ECs were gated as single, live, CD45$^-$ CD11b$^-$ Ly6C$^+$ CD31$^+$ cells. (B) Quantification of eYFP$^+$ CNS ECs in A20$^{\Delta CNS\text{-}EC}$-eYFP mice compared to A20$^{fl/fl}$ controls (n = 4-6 mice per group). (C) Representative mapping of RNA-seq reads to *Tnfaip3* locus confirming excision of exon 3 in A20$^{\Delta CNS\text{-}EC}$ mice. (D) Analysis of Gene Ontology (GO) terms of Biological Processes from the 30 mutually DE genes shown in Figure 5D. (E) Scatter plot showing Log2 fold changes in EAE vs. control against A20$^{\Delta CNS\text{-}EC}$-eYFP vs. control of all genes. *Icam1*, *Vcam1* and *Icosl* are highlighted in bigger red circles. (F) RT-PCR of *Icosl* in sorted ECs from A20$^{\Delta CNS\text{-}EC}$-eYFP and A20$^{fl/fl}$ mice (n = 3). (G) pMBMECs from A20$^{fl/fl}$ and A20$^{\Delta CNS\text{-}EC}$ mice were stimulated with 10 ng/ml TNF for 6 h and *Icosl* mRNA levels were determined by RT-PCR. Representative of 2 individual experiments with n = 3-4 per group. (H) pMBMECs from wildtype mice were stimulated with 10 ng/ml TNF or 10 ng/ml IL-1β for 6h and *Icosl* expression levels were determined by RT-PCR. Representative of 3 individual experiments with n = 3 per group. *Icosl* levels in F-H are presented relative to controls. (I) pMBMECs from wildtype mice were stimulated with 10 ng/ml IL-1β for 48 or 72h. ICOSL, ICAM-1 and VCAM-1 protein expression levels were determined by flow cytometry. Data is representative for 2 individual experiments with n = 3-4 per group. Statistical significance was determined by two-tailed unpaired Student's t-test (B, F), ordinary one-way ANOVA with Tukey's multiple comparisons test (G, H) or mixed-effects model with Geisser-Greenhouse correction and Holm-Šidák multiple comparison test (I). * $p<0.05$, ** $p<0.01$, *** $p<0.001$, **** $p<0.0001$.

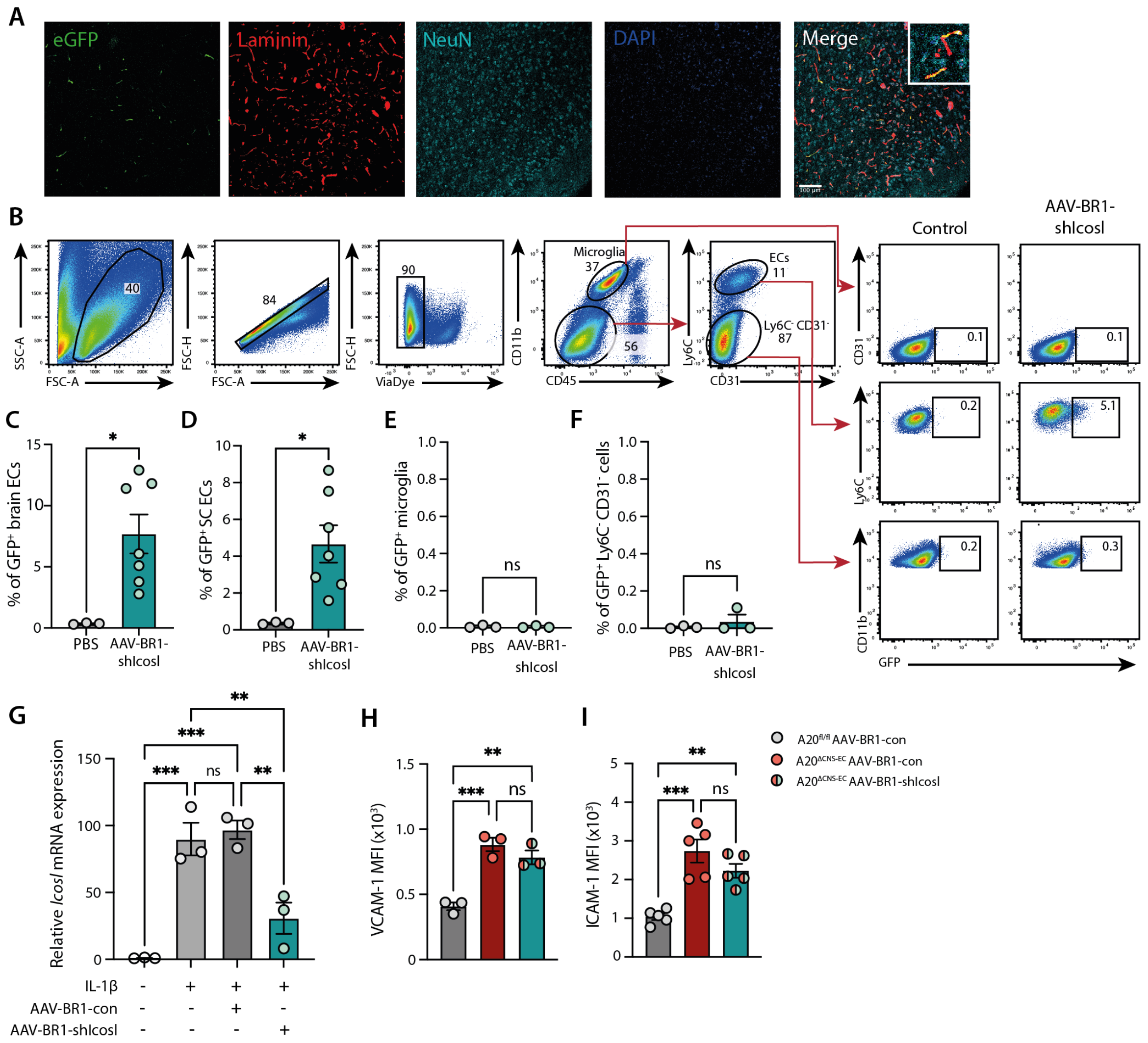

Supp. Figure 6: AAV-BR1-shIcosl specifically targets CNS-ECs and efficiently knocks down ICOSL

(A) Representative immunofluorescent staining of brain tissue from wildtype mice treated with 1.8×10^{11} gp AAV-BR1-eGFP and two weeks later immunized with MOG/CFA + PTx. Tissue was collected at the peak of the disease (DPI15) and stained for NeuN (cyan) and laminin (red) Endogenous eGFP is shown in green and nuclear staining in blue (DAPI). Scale bar = 100 µm. (n=3). (B-F) Flow cytometry analysis of GFP$^+$ cells in the CNS of wildtype mice four weeks after i.v. injection of AAV-BR1-shIcosl encoding for a GFP under the RSV promoter or PBS as control. Data is representative for 3 individual experiments with n = 3-7 mice per group. (A) Representative gating strategy for microglia as CD45^{int} CD11b$^+$ cells, for CD45$^-$ CD11b$^-$ Ly6C$^-$ CD31$^-$ cells and for CD45$^-$ CD11b$^-$ Ly6C$^+$ CD31$^+$ CNS ECs. (C) Quantification of GFP$^+$ CNS ECs in the brain and (D) in the SC. (E) Quantification of GFP$^+$ microglia and (F) of GFP$^+$ Ly6C$^-$ CD31$^-$ cells. (G) pMBMECs from wildtype mice were treated with 0.6×10^6 gp of AAV-BR1-shIcosl or AAV-BR1-con four days after seeding. Three days after transfection cells were stimulated with 10 ng/mL IL-1β for 6h. *Icosl* expression levels were determined by RT-PCR and are presented relative to unstimulated control. Data is representative for 2 individual experiments with n = 3 per group. (H-I) Flow cytometry analysis of VCAM-1 and ICAM-1 in steady state CNS-ECs from A20$^{\Delta CNS\text{-}EC}$ and A20$^{fl/fl}$ mice four weeks after i.v. injection of AAV-BR1-shIcosl or AAV-BR1-con. Mean fluorescence intensity (MFI) of VCAM-1 (H) and ICAM-1 (I) were quantified as geometric means (n = 3-5 mice per group). Statistical significance was determined by two-tailed unpaired Student's t-test (C-F) or ordinary one-way ANOVA with Tukey's multiple comparisons test (G-I). * p<0.05, ** p<0.01, *** p<0.001, ns = not significant.
